# Supplementary material for: Distinct melanocyte subpopulations defined by stochastic expression of proliferation or maturation programs enable a rapid and sustainable pigmentation response
Source: PLoS Biol. 2024 Aug 20;22(8):e3002776. doi: 10.1371/journal.pbio.3002776 (PMC11364419; doi:10.1371/journal.pbio.3002776)
Supplement: S1 Fig — (A) UMAP visualisation of the human skin melanocyte coloured by age group (top), donor ID (bottom left), plate ID (bottom right). (B) UMAP visualisation of the human skin melanocyte coloured by the clusters. (C) Dot plot showing the top 10 marker genes enriched in each cluster, with size depicting the percent cells expressing the gene and colour depicting the scaled mean expression value in each cluster (Wilcoxon–Mann–Whitney test with average log fold change >0.25 and adjusted p-value ≤0.05). (D) Dot plot depicting top 5 GO terms enriched in each cluster identified using cluster markers. (E) Stacked bar plot showing the distribution of different age groups in each of the identified cell states. All numerical data are listed in S1 Data. (DOCX) [file pbio.3002776.s001.docx]

**Supporting Information for**

**Distinct melanocyte subpopulations defined by stochastic expression of proliferation or maturation programs enable a rapid and sustainable Pigmentation response**

Ayush Aggarwal^1,2^, Ayesha Nasreen^1,2^, Babita Sharma^1,2^, Sarthak Sahoo^3^, Keerthic Aswin^1,2^, Mohammed Faruq^1,2^, Rajesh Pandey^1,2^, Mohit K Jolly^3^, Abhyudai Singh^4,5^, Rajesh S Gokhale^6,7^ and Vivek T Natarajan^1,2*^

Vivek T Natarajan, PhD

CSIR-Institute of Genomics and Integrative Biology

Mathura Road, Delhi 110 020, India

Phone No. 91-011-29879203

**Email:**  [tnvivek@igib.in,](mailto:tnvivek@igib.in,) [tnvivek@igib.res.in](mailto:tnvivek@igib.res.in)


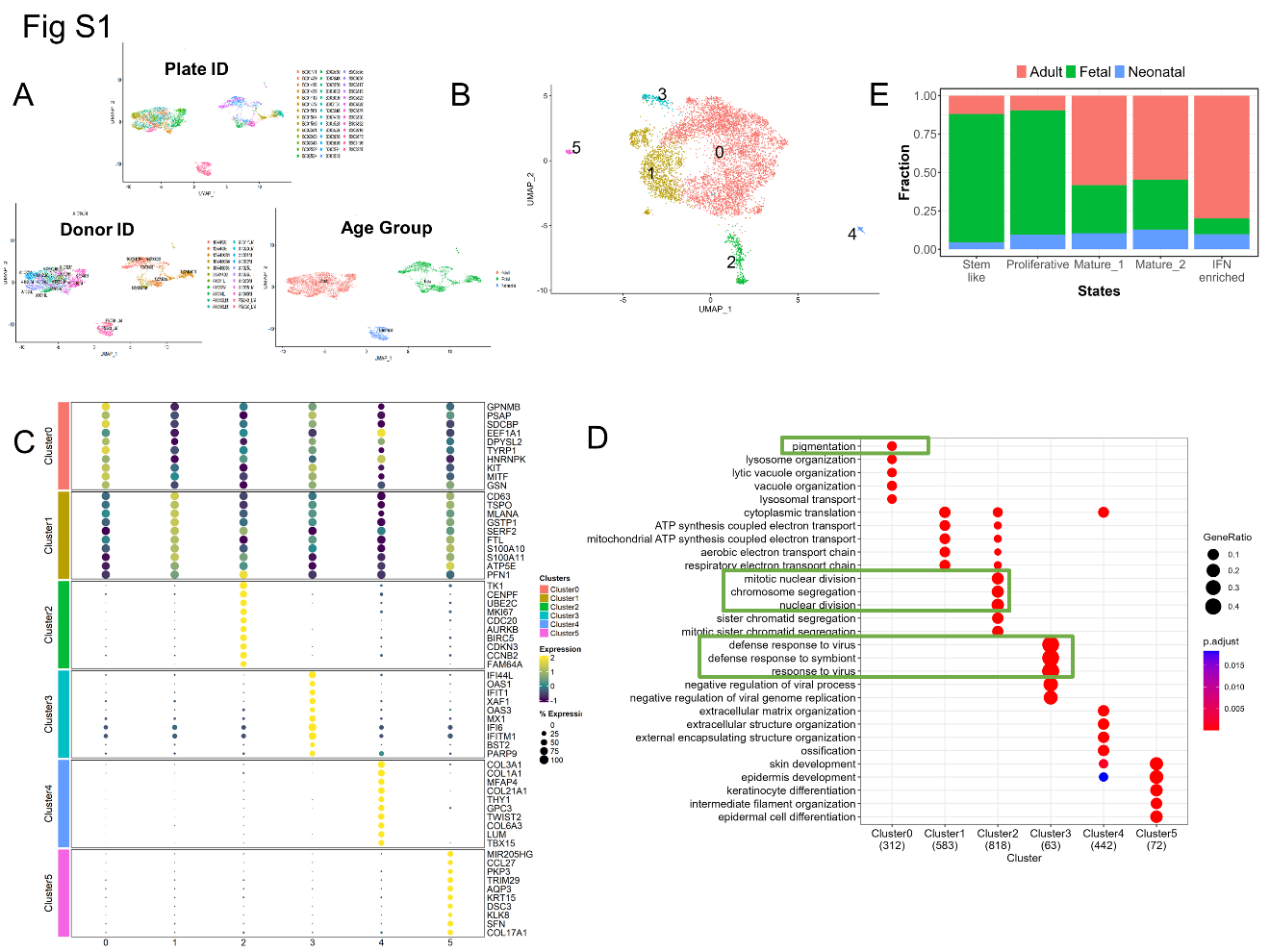


**Fig S1: Analysis of epidermal NHEM scRNA seq data taken from GSE151091 (related to Fig 1)**

1. UMAP visualization of the human skin melanocyte coloured by age group (top), donor ID (bottom left), plate ID (bottom right)
2. UMAP visualization of the human skin melanocyte coloured by the clusters
3. Dot plot showing the top 10 marker genes enriched in each cluster, with size depicting the percent cells expressing the gene and colour depicting the scaled mean expression value in each cluster (Wilcoxon-Mann-Whitney test with average log fold change > 0.25 and adjusted p value ≤ 0.05).
4. Dot plot depicting top 5 GO terms enriched in each cluster identified using cluster markers
5. Stacked bar plot showing the distribution of different age groups in each of the identified cell states.

All numerical data are listed in S1 data.
