# Supplementary material for: Distinct melanocyte subpopulations defined by stochastic expression of proliferation or maturation programs enable a rapid and sustainable pigmentation response
Source: PLoS Biol. 2024 Aug 20;22(8):e3002776. doi: 10.1371/journal.pbio.3002776 (PMC11364419; doi:10.1371/journal.pbio.3002776)
Supplement: S2 Fig — (A) UMAP visualisation of the human skin melanocyte coloured by the clusters. (B) Dot plot of depicting the top 10 marker genes enriched in each cluster with size showing the percent cells expressing the gene and colour depicting the scaled mean expression value in each cluster (Wilcoxon–Mann–Whitney test with average log fold change > 0.25 and adjusted p-value ≤0.05). (C) Dot plot depicting top 5 GO terms enriched in each cluster identified using cluster markers. (D) Heat map depicting the gene-set activity scores (z-score of mean) of each of the epidermal NHEM clusters for the human melanocyte mature, proliferative, IFN enriched, and stem-like state. (E) Heatmap depicting the scores (z-score of mean) of each of the B16 clusters for the mouse hair follicle melanocyte mature, proliferative and stem-like state. All numerical data are listed in S1 Data. (DOCX) [file pbio.3002776.s002.docx]

**Supporting Information for**

**Distinct melanocyte subpopulations defined by stochastic expression of proliferation or maturation programs enable a rapid and sustainable Pigmentation response**

Ayush Aggarwal^1,2^, Ayesha Nasreen^1,2^, Babita Sharma^1,2^, Sarthak Sahoo^3^, Keerthic Aswin^1,2^, Mohammed Faruq^1,2^, Rajesh Pandey^1,2^, Mohit K Jolly^3^, Abhyudai Singh^4,5^, Rajesh S Gokhale^6,7^ and Vivek T Natarajan^1,2*^

Vivek T Natarajan, PhD

CSIR-Institute of Genomics and Integrative Biology

Mathura Road, Delhi 110 020, India

Phone No. 91-011-29879203

**Email:**  [tnvivek@igib.in,](mailto:tnvivek@igib.in,) [tnvivek@igib.res.in](mailto:tnvivek@igib.res.in)


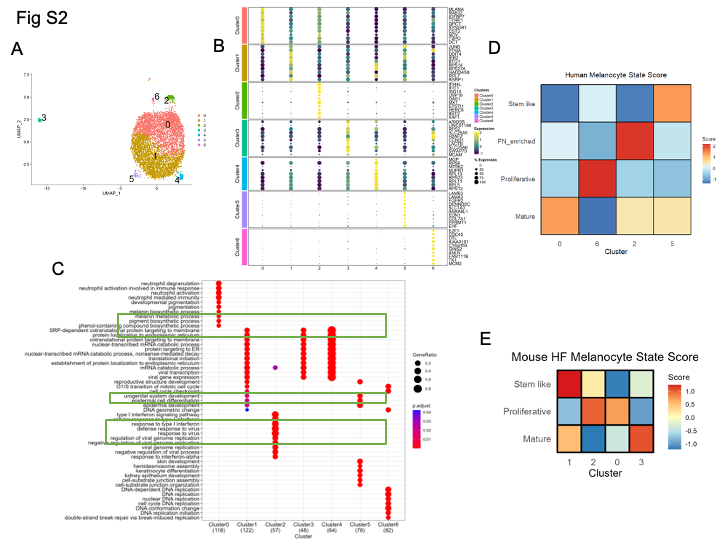


Fig S2: Analysis of epidermal NHEM scRNA seq data taken from EGAS00001002927 and mouse Hair follicle dataset (GSE147299) (related to Fig 1 and Fig 4 respectively)

1. UMAP visualization of the human skin melanocyte coloured by the clusters
2. Dot plot of depicting the top 10 marker genes enriched in each cluster with size showing the percent cells expressing the gene and colour depicting the scaled mean expression value in each cluster (Wilcoxon-Mann-Whitney test with average log fold change > 0.25 and adjusted p value ≤ 0.05).
3. Dot plot depicting top 5 GO terms enriched in each cluster identified using cluster markers
4. Heat map depicting the gene-set activity scores (z-score of mean) of each of the epidermal NHEM clusters for the human melanocyte mature, proliferative, IFN enriched and Stem like state.
5. Heatmap depicting the scores (z-score of mean) of each of the B16 clusters for the mouse hair follicle melanocyte mature, proliferative and stem like state.

All numerical data are listed in S1 data.
