# Supplementary material for: Distinct melanocyte subpopulations defined by stochastic expression of proliferation or maturation programs enable a rapid and sustainable pigmentation response
Source: PLoS Biol. 2024 Aug 20;22(8):e3002776. doi: 10.1371/journal.pbio.3002776 (PMC11364419; doi:10.1371/journal.pbio.3002776)
Supplement: S3 Fig — (A) Immunofluorescence images of NHEMs treated either daily or every alternate day with forskolin and stained for proliferative and pigmenting marker proteins. Nuclear DNA stained with DAPI (blue), Ki67 and TYR in green. Scale bars represent 150 μm. (B) Quantitation of corrected total cell fluorescence (CTCF) of individual cells for Ki67 and TYR in daily and alternate day forskolin-treated NHEMs. p-value via an unpaired, two-tailed Student’s t test, with significant values (p < 0.05) is displayed on the graph. (C) Western blot analysis of daily and alternate day forskolin-treated NHEM cells. Numbers below the blot represents fold change wrt daily forskolin-treated cells. (DF) Daily forskolin, (AF) alternate forskolin-treated NHEMs. (DOCX) [file pbio.3002776.s003.docx]

**Supporting Information for**

**Distinct melanocyte subpopulations defined by stochastic expression of proliferation or maturation programs enable a rapid and sustainable Pigmentation response**

Ayush Aggarwal^1,2^, Ayesha Nasreen^1,2^, Babita Sharma^1,2^, Sarthak Sahoo^3^, Keerthic Aswin^1,2^, Mohammed Faruq^1,2^, Rajesh Pandey^1,2^, Mohit K Jolly^3^, Abhyudai Singh^4,5^, Rajesh S Gokhale^6,7^ and Vivek T Natarajan^1,2*^

Vivek T Natarajan, PhD

CSIR-Institute of Genomics and Integrative Biology

Mathura Road, Delhi 110 020, India

Phone No. 91-011-29879203

**Email:**  [tnvivek@igib.in,](mailto:tnvivek@igib.in,) [tnvivek@igib.res.in](mailto:tnvivek@igib.res.in)


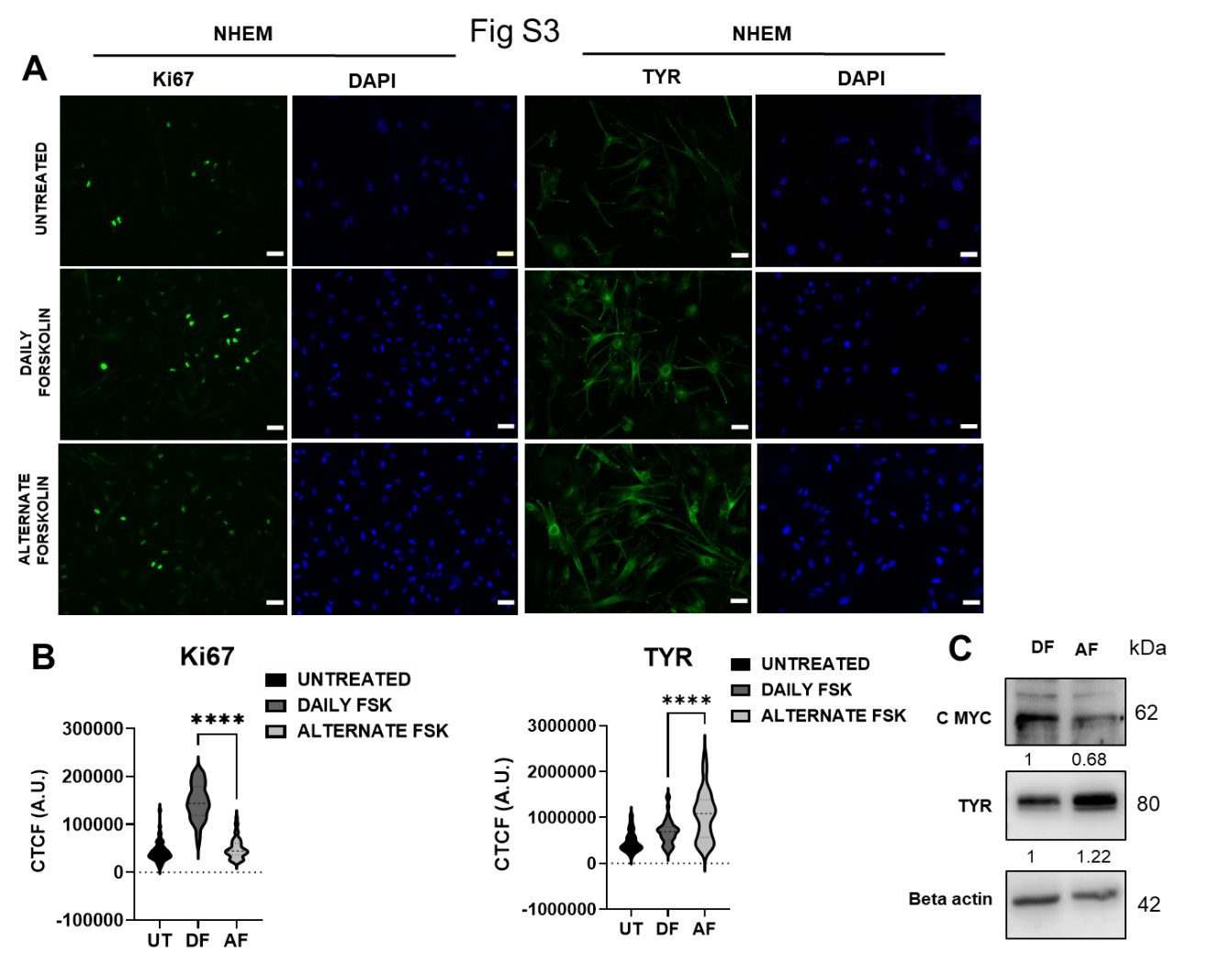
**Fig S3: Immunofluorescence and western blot analysis of daily and alternate day Forskolin treated NHEMs.**

1. Immunofluorescence images of NHEMs treated either daily or every alternate day with forskolin and stained for proliferative and pigmenting marker proteins. Nuclear DNA stained with DAPI (blue), Ki67 and TYR in green. Scale bars represent 150 μm.
2. Quantitation of corrected total cell fluorescence (CTCF) of individual cells for Ki67 and TYR in daily and alternate day Forskolin treated NHEMs. *p*-value via an unpaired, two-tailed Student’s *t*-test, with significant values (*p* < 0.05) is displayed on the graph.
3. Western blot analysis of daily and alternate day forskolin treated NHEM cells. Numbers below the blot represents fold change *wrt* daily forskolin treated cells. (DF) Daily Forskolin, (AF) Alternate Forskolin treated NHEMs.
