# Supplementary material for: Distinct melanocyte subpopulations defined by stochastic expression of proliferation or maturation programs enable a rapid and sustainable pigmentation response
Source: PLoS Biol. 2024 Aug 20;22(8):e3002776. doi: 10.1371/journal.pbio.3002776 (PMC11364419; doi:10.1371/journal.pbio.3002776)
Supplement: S4 Fig — (A) Immunofluorescence images of MNT-1 cells treated either daily or every alternate day with forskolin and stained for proliferative and pigmenting marker proteins. Nuclear DNA stained with DAPI (blue), Ki67 and TYR in green. Scale bars represent 150 μm. Quantitation of corrected total cell fluorescence (CTCF) of individual cells for Ki67 and TYR in daily and alternate day forskolin-treated MNT-1 cells. p-value via an unpaired, two-tailed Student’s t test, with significant values (p < 0.05) is displayed on the graph. (B) Western blot analysis of c-MYC and TYR in daily and alternate day forskolin-treated MNT-1 cells. Numbers below the blot represents fold change wrt daily forskolin-treated cells. (C) Quantitation of percent Ki67/TYR positive cells from 2 biological replicates of MNT-1 with daily or alternate day forskolin treatment. Two-way ANOVA was performed. Adjusted P-values: ** P-value <0.001, *** P-value <0.0001, **** P-value <0.00001. (D) Quantitation of mean fluorescence intensity per cell from 2 biological replicates of MNT-1 with daily or alternate day forskolin treatment. Two-way ANOVA was performed. Adjusted P-values: ** P-value <0.001, *** P-value <0.0001, **** P-value <0.00001. (DOCX) [file pbio.3002776.s004.docx]

**Supporting Information for**

**Distinct melanocyte subpopulations defined by stochastic expression of proliferation or maturation programs enable a rapid and sustainable Pigmentation response**

Ayush Aggarwal^1,2^, Ayesha Nasreen^1,2^, Babita Sharma^1,2^, Sarthak Sahoo^3^, Keerthic Aswin^1,2^, Mohammed Faruq^1,2^, Rajesh Pandey^1,2^, Mohit K Jolly^3^, Abhyudai Singh^4,5^, Rajesh S Gokhale^6,7^ and Vivek T Natarajan^1,2*^

Vivek T Natarajan, PhD

CSIR-Institute of Genomics and Integrative Biology

Mathura Road, Delhi 110 020, India

Phone No. 91-011-29879203

**Email:**  [tnvivek@igib.in,](mailto:tnvivek@igib.in,) [tnvivek@igib.res.in](mailto:tnvivek@igib.res.in)


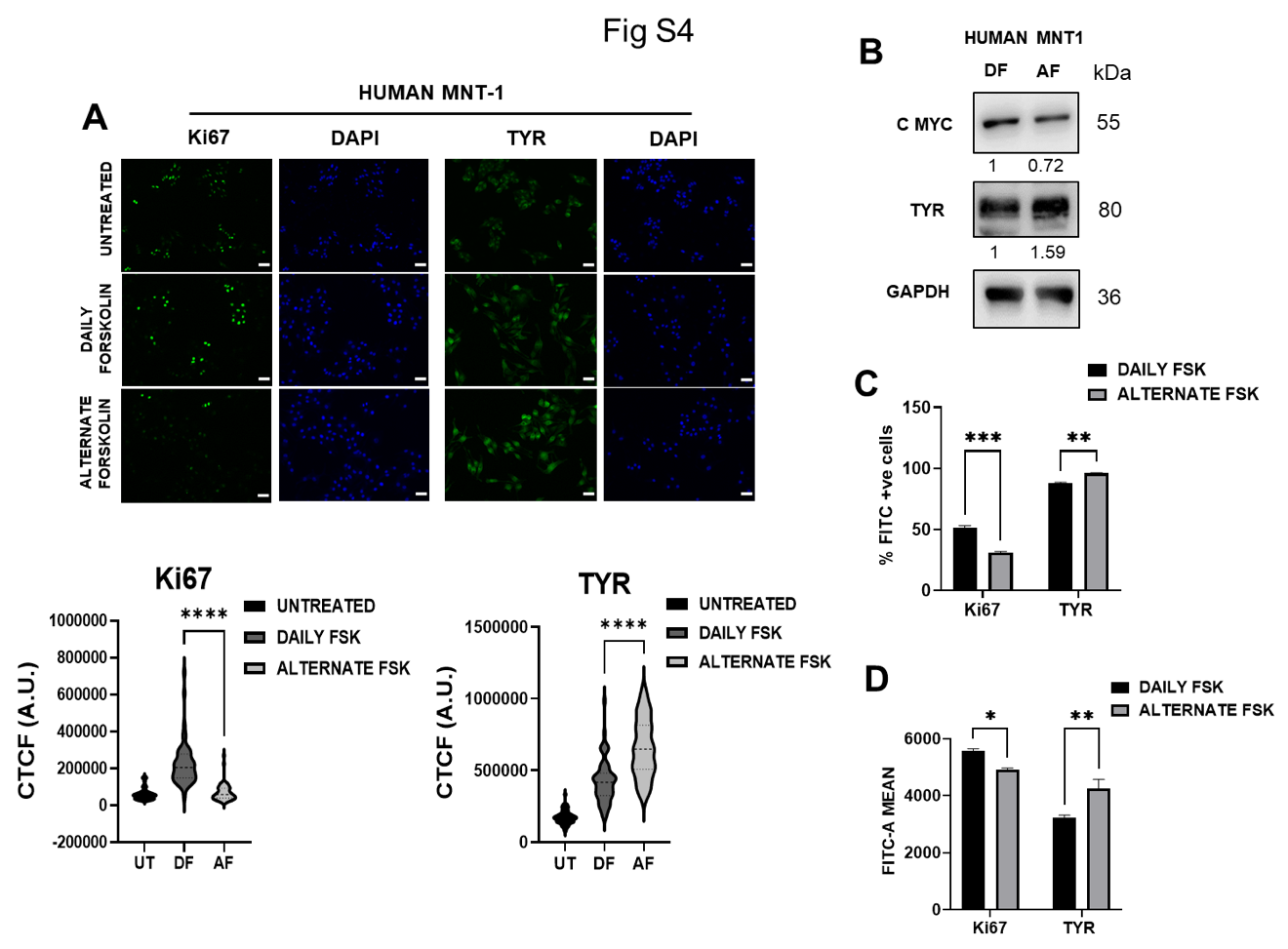


**Fig S4: Immunofluorescence, western blot and FACS analysis of daily and alternate day Forskolin treated human MNT-1 cells.**

1. Immunofluorescence images of MNT-1 cells treated either daily or every alternate day with forskolin and stained for proliferative and pigmenting marker proteins. Nuclear DNA stained with DAPI (blue), Ki67 and TYR in green. Scale bars represent 150 μm. Quantitation of Corrected total cell fluorescence (CTCF) of individual cells for Ki67 and TYR in daily and alternate day Forskolin treated MNT-1 cells. *p*-value via an unpaired, two-tailed Student’s *t*-test, with significant values (*p* < 0.05) is displayed on the graph.
2. Western blot analysis of c-MYC and TYR in daily and alternate day Forskolin treated MNT-1 cells. Numbers below the blot represents fold change *wrt* daily forskolin treated cells.
3. Quantitation of percent Ki67/TYR positive cells from two biological replicates of MNT-1 with daily or alternate day forskolin treatment. Two-way ANOVA was performed. Adjusted *P* values: ** *P*-value < 0.001, *** *P*-value < 0.0001, **** *P*-value < 0.00001.
4. Quantitation of mean fluorescence intensity per cell from two biological replicates of MNT-1 with daily or alternate day forskolin treatment. Two-way ANOVA was performed. Adjusted *P* values: ** *P*-value < 0.001, *** *P*-value < 0.0001, **** *P*-value < 0.00001.
