# Supplementary material for: Distinct melanocyte subpopulations defined by stochastic expression of proliferation or maturation programs enable a rapid and sustainable pigmentation response
Source: PLoS Biol. 2024 Aug 20;22(8):e3002776. doi: 10.1371/journal.pbio.3002776 (PMC11364419; doi:10.1371/journal.pbio.3002776)
Supplement: S5 Fig — (A) Bright field images of day 7 B16 low and high pigmenting colonies. The bottom panel represents images of single cells sorted using imaging flow cytometry from the differentially pigmented day 7 sample. Scale bars are indicated. (B) Pearson’s correlation analysis between the average melanin content per cell estimated using NaOH method and the different parameters of imaging flow cytometer. (C) Linear regression analysis of mean brightfield intensity of imaging flow cytometer and average melanin content per cell estimated using NaOH method. (D) Distribution of pigmentation in B16 cells at day 7 of the progressive pigmentation model. (E) Side scatter intensity distribution (representing pigmentation) of B16 cells at days 0 and 7 of the progressive pigmentation model. Rectangle gate represents high pigment cells. All numerical data are listed in S1 Data. (DOCX) [file pbio.3002776.s005.docx]

**Supporting Information for**

**Distinct melanocyte subpopulations defined by stochastic expression of proliferation or maturation programs enable a rapid and sustainable Pigmentation response**

Ayush Aggarwal^1,2^, Ayesha Nasreen^1,2^, Babita Sharma^1,2^, Sarthak Sahoo^3^, Keerthic Aswin^1,2^, Mohammed Faruq^1,2^, Rajesh Pandey^1,2^, Mohit K Jolly^3^, Abhyudai Singh^4,5^, Rajesh S Gokhale^6,7^ and Vivek T Natarajan^1,2*^

Vivek T Natarajan, PhD

CSIR-Institute of Genomics and Integrative Biology

Mathura Road, Delhi 110 020, India

Phone No. 91-011-29879203

**Email:**  [tnvivek@igib.in,](mailto:tnvivek@igib.in,) [tnvivek@igib.res.in](mailto:tnvivek@igib.res.in)


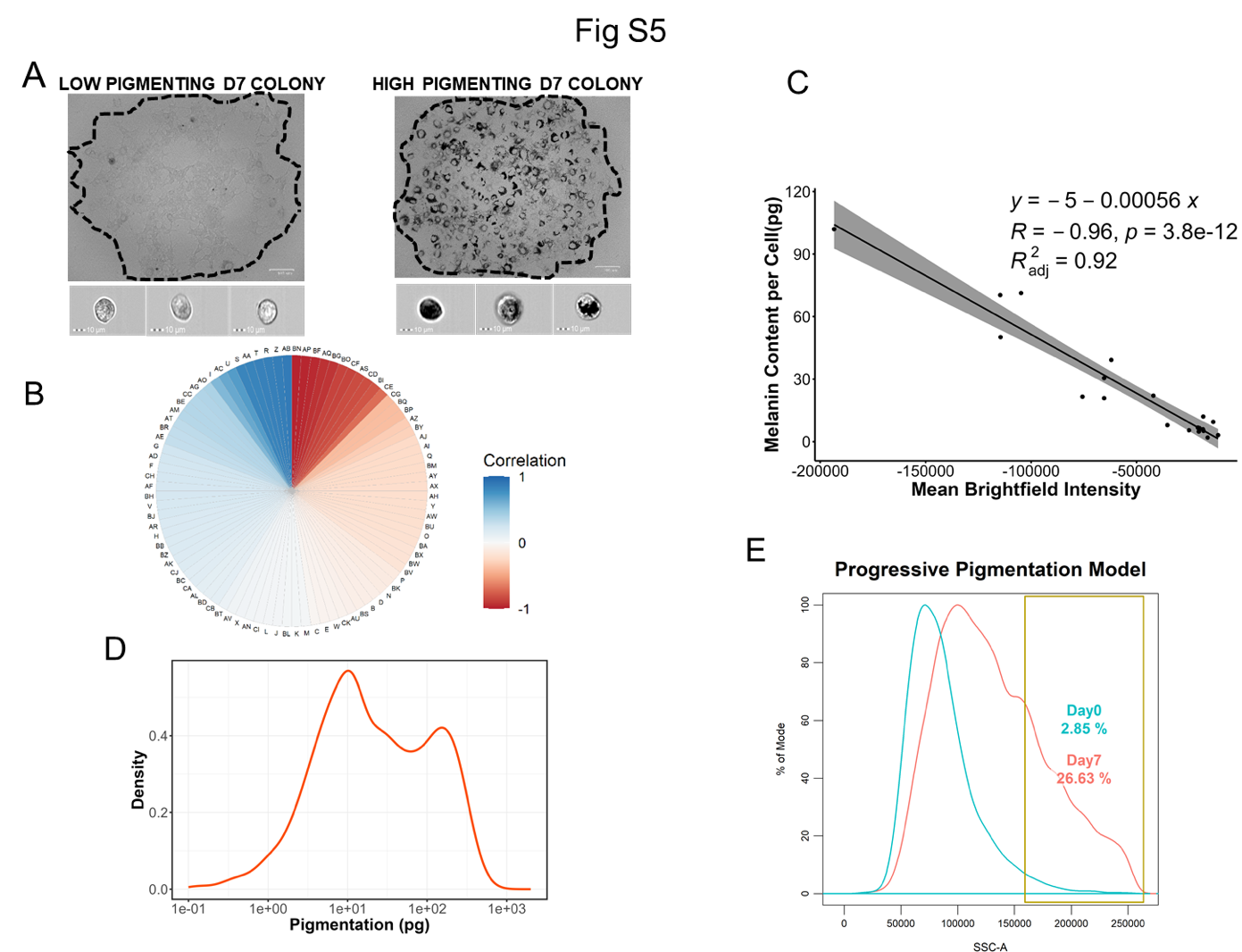


Fig S5: Assessment of low and high pigmentation melanocyte states (related to Fig 2)

1. Bright field images of day7 B16 low and high pigmenting colonies. The bottom panel represents images of single cells sorted using imaging flow cytometry from the differentially pigmented day 7 sample. Scale bars is indicated.
2. Pearson’s correlation analysis between the average melanin content per cell estimated using NaOH method and the different parameters of imaging flow cytometer.
3. Linear regression analysis of mean brightfield intensity of imaging flow cytometer and average melanin content per cell estimated using NaOH method.
4. Distribution of pigmentation in B16 cells at day 7 of the progressive pigmentation model.
5. Side scatter intensity distribution (representing pigmentation) of B16 cells at day0 and 7 of the progressive pigmentation model. Rectangle gate represents high pigment cells.

All numerical data are listed in S1 data.
