# Supplementary material for: Distinct melanocyte subpopulations defined by stochastic expression of proliferation or maturation programs enable a rapid and sustainable pigmentation response
Source: PLoS Biol. 2024 Aug 20;22(8):e3002776. doi: 10.1371/journal.pbio.3002776 (PMC11364419; doi:10.1371/journal.pbio.3002776)
Supplement: S6 Fig — (A) Immunofluorescence images of day 7 B16 cells depicting low (LP) and high (HP) pigmenting colonies that were stained for proliferative and pigmenting marker proteins. Nuclear DNA stained with DAPI (blue), Ki67 (green), and TYR (red). Scale bars represent 150 μm. (B) Quantitation of corrected total cell fluorescence (CTCF) of individual cells for Ki67 and TYR in low and high pigmenting B16 cells. (MEL LOW) low melanin, (MEL HIGH) high melanin content based on visual inspection in bright field image. p-value via an unpaired, two-tailed Student’s t test, with significant values (p < 0.05) is displayed on the graph. (C) Western blot images and quantitation of protein levels of TWIST 1, C MYC, LEF 1, TYR, and DCT in B16 day 7 cells sorted based on pigmentation. Quantitation with respect to low pigmenting cells is depicted below the blot images. (LP) Low pigmenting, (HP) high pigmenting B16 cells sorted based on FACS using side scatter information. Experiments were performed in duplicates. (DOCX) [file pbio.3002776.s006.docx]

**Supporting Information for**

**Distinct melanocyte subpopulations defined by stochastic expression of proliferation or maturation programs enable a rapid and sustainable Pigmentation response**

Ayush Aggarwal^1,2^, Ayesha Nasreen^1,2^, Babita Sharma^1,2^, Sarthak Sahoo^3^, Keerthic Aswin^1,2^, Mohammed Faruq^1,2^, Rajesh Pandey^1,2^, Mohit K Jolly^3^, Abhyudai Singh^4,5^, Rajesh S Gokhale^6,7^ and Vivek T Natarajan^1,2*^

Vivek T Natarajan, PhD

CSIR-Institute of Genomics and Integrative Biology

Mathura Road, Delhi 110 020, India

Phone No. 91-011-29879203

**Email:**  [tnvivek@igib.in,](mailto:tnvivek@igib.in,) tnvivek@igib.res.in


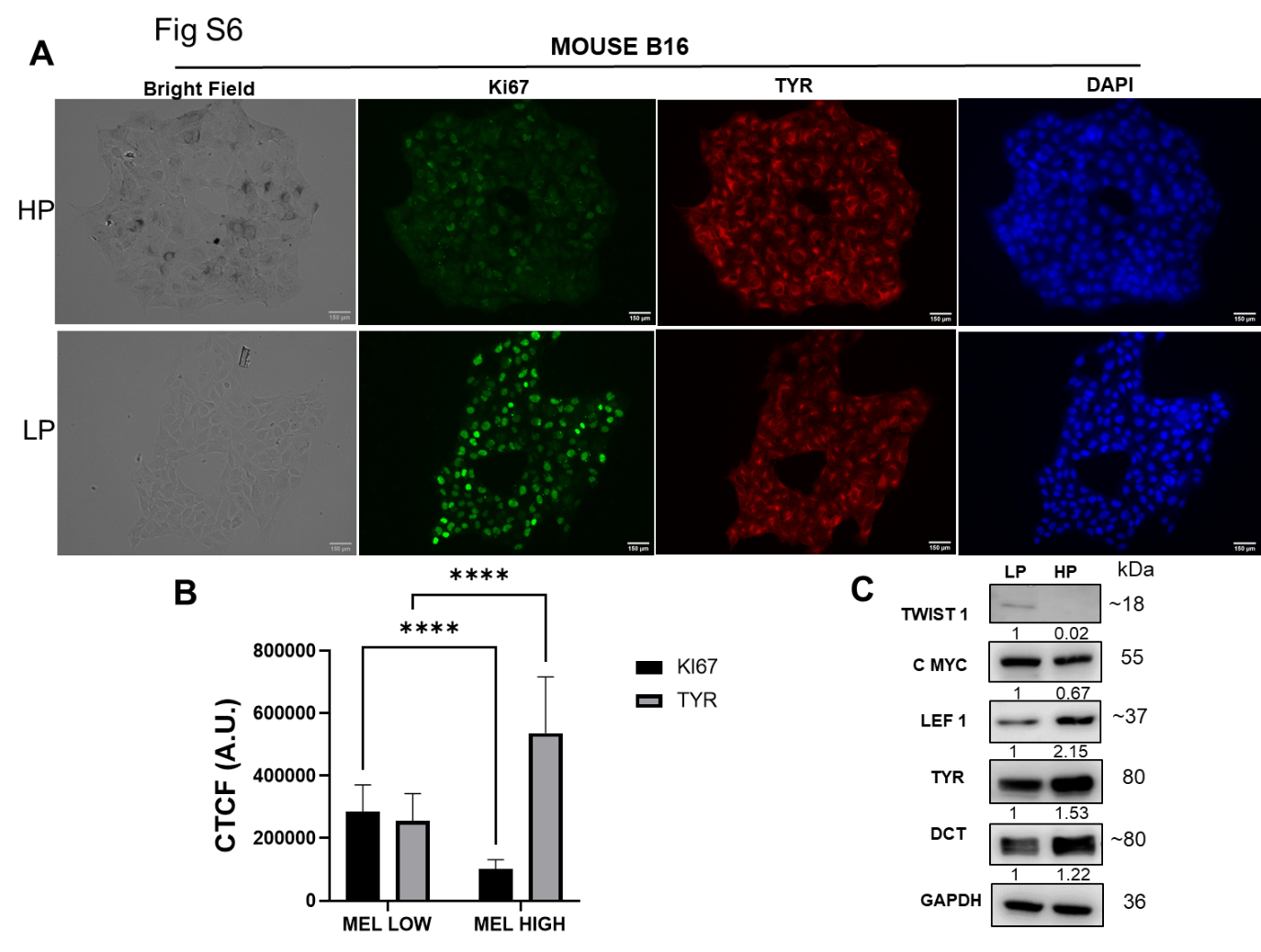


Fig S6: Immunofluorescence and western blot based analysis of differentially pigmented B16 mouse melanoma cells

1. Immunofluorescence images of day 7 B16 cells depicting low (LP) and high (HP) pigmenting colonies that were stained for proliferative and pigmenting marker proteins. Nuclear DNA stained with DAPI (blue), Ki67 (green) and TYR (red). Scale bars represent 150 μm.
2. Quantitation of corrected total cell fluorescence (CTCF) of individual cells for Ki67 and TYR in low and high pigmenting B16 cells. (MEL LOW) low melanin, (MEL HIGH) high melanin content based on visual inspection in bright field image. *p*-value via an unpaired, two-tailed Student’s *t*-test, with significant values (*p* < 0.05) is displayed on the graph.
3. Western blot images and quantitation of protein levels of TWIST 1, C MYC, LEF 1, TYR and DCT in B16 day 7 cells sorted based on pigmentation. Quantitation with respect to low pigmenting cells is depicted below the blot images. (LP) Low Pigmenting, (HP) High Pigmenting B16 cells sorted based on FACS using side scatter information. Experiments were performed in duplicates.
