# Supplementary material for: Distinct melanocyte subpopulations defined by stochastic expression of proliferation or maturation programs enable a rapid and sustainable pigmentation response
Source: PLoS Biol. 2024 Aug 20;22(8):e3002776. doi: 10.1371/journal.pbio.3002776 (PMC11364419; doi:10.1371/journal.pbio.3002776)
Supplement: S7 Fig — (A) Density plot showing the distribution of pigmentation (log scale) in single cells across differentially pigmented B16 samples. Inset: statistical analysis of mean pigmentation, Wilcoxon–Mann–Whitney test, n = 3, ****: p-value ≤ 0.0001, ns: p-value >0.05. High pigment: 50 μm IBMX, low pigment: 100 μm PTU. (B) Density plot showing the distribution of pigmentation (log scale) in single colonies at day 7 of the pigmentation model setup using the B16 samples in panel E. Inset: statistical analysis of mean pigmentation, Wilcoxon–Mann–Whitney test, ****: p-value ≤0.0001, ns: p-value >0.05. (C) Distribution of pigmentation in B16 colonies at day 7 of the progressive pigmentation model. (D) Side scatter intensity distribution (representing pigmentation) of B16 cells at days 0, 3, and 5 of the progressive pigmentation model. Rectangle gate represents high pigment cells. (E) Dot plot showing the top 15 marker genes enriched in each subcluster of day 0 B16 cells with the size showing the percent of cell expressing the gene and colour showing the scaled mean expression value in each cluster (Wilcoxon–Mann–Whitney test with average log fold change >0.2 and adjusted p-value ≤0.05). (F) Distribution of ATAC peaks around the TSS in different melanocyte states of the progressive pigmentation model. (G) Side scatter intensity distribution (representing pigmentation) of B16 cells at days 0 and 5 of the progressive pigmentation model. Rectangle gate represents high pigment cells. All numerical data are listed in S1 Data. (DOCX) [file pbio.3002776.s007.docx]

**Supporting Information for**

**Distinct melanocyte subpopulations defined by stochastic expression of proliferation or maturation programs enable a rapid and sustainable Pigmentation response**

Ayush Aggarwal^1,2^, Ayesha Nasreen^1,2^, Babita Sharma^1,2^, Sarthak Sahoo^3^, Keerthic Aswin^1,2^, Mohammed Faruq^1,2^, Rajesh Pandey^1,2^, Mohit K Jolly^3^, Abhyudai Singh^4,5^, Rajesh S Gokhale^6,7^ and Vivek T Natarajan^1,2*^

Vivek T Natarajan, PhD

CSIR-Institute of Genomics and Integrative Biology

Mathura Road, Delhi 110 020, India

Phone No. 91-011-29879203

**Email:**  [tnvivek@igib.in,](mailto:tnvivek@igib.in,) [tnvivek@igib.res.in](mailto:tnvivek@igib.res.in)


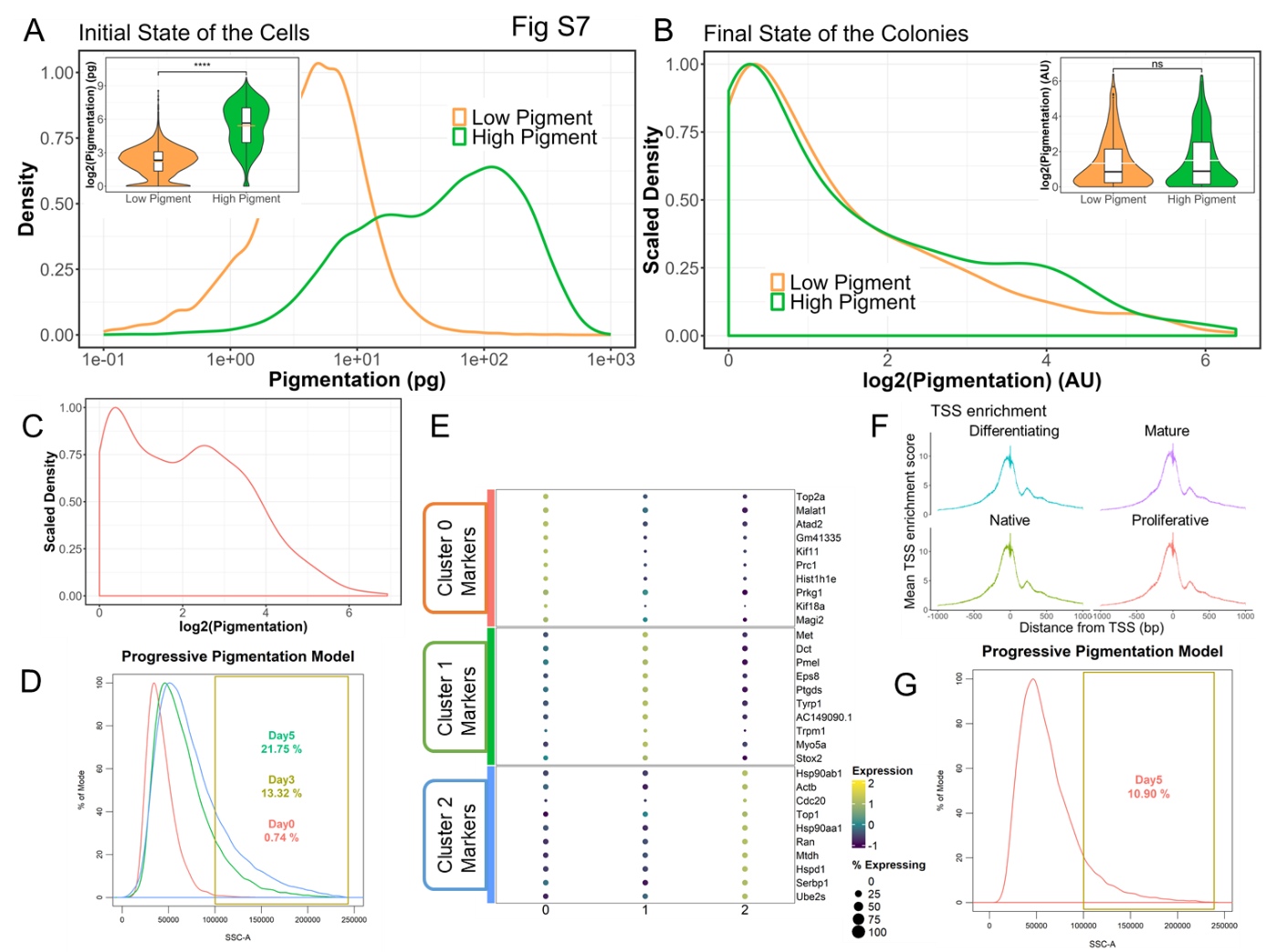


Fig S7: Imaging based assessment of differentially pigmented B16 states and quality control of scMultiomics data (related to Fig 3 and 4)

1. Density plot showing the distribution of pigmentation (log scale) in single cells across differentially pigmented B16 samples. Inset: statistical analysis of mean pigmentation, Wilcoxon-Mann-Whitney test, n=3, ****: p value ≤ 0.0001, ns: p value > 0.05. High Pigment: 50µM IBMX, Low Pigment: 100µM PTU.
2. Density plot showing the distribution of pigmentation (log scale) in single colonies at day 7 of the pigmentation model setup using the B16 samples in panel E. Inset: statistical analysis of mean pigmentation, Wilcoxon-Mann-Whitney test, ****: p value ≤ 0.0001, ns: p value > 0.05.
3. Distribution of pigmentation in B16 colonies at day 7 of the progressive pigmentation model.
4. Side scatter intensity distribution (representing pigmentation) of B16 cells at day0, 3 and 5 of the progressive pigmentation model. Rectangle gate represents high pigment cells.
5. Dot plot showing the top 15 marker genes enriched in each sub-cluster of day 0 B16 cells with the size showing the percent of cell expressing the gene and colour showing the scaled mean expression value in each cluster (Wilcoxon-Mann-Whitney test with average log fold change > 0.2 and adjusted p value ≤ 0.05).
6. Distribution of ATAC peaks around the TSS in different melanocyte states of the progressive pigmentation model.
7. Side scatter intensity distribution (representing pigmentation) of B16 cells at day0 and 5 of the progressive pigmentation model. Rectangle gate represents high pigment cells.

All numerical data are listed in S1 data.
