# Supplementary material for: Distinct melanocyte subpopulations defined by stochastic expression of proliferation or maturation programs enable a rapid and sustainable pigmentation response
Source: PLoS Biol. 2024 Aug 20;22(8):e3002776. doi: 10.1371/journal.pbio.3002776 (PMC11364419; doi:10.1371/journal.pbio.3002776)
Supplement: S8 Fig — (A) Plots showing distribution of H3K27ac peaks around the TSS (top), annotation of peaks (bottom left), and distribution of peaks relative to TSS in day 5 LP and HP population. (B) UMAP plot showing day 7 cells of the progressive pigmentation model coloured by clusters identified using TF activity (top) and pigmentation (bottom). (C) Heatmap showing top TF active in LP and HP population at day 7 (right). TF activity derived from scRNA data using Dorothea and Viper packages. (D) PCA plot showing expression of Myc (top), E2f (middle), and pigmentation genes (bottom) in the artificial cells simulated using RACIPE. (E) Violin plot showing Mitf expression in differentiating, mature, native, and proliferative melanocyte states in the progressive pigmentation model. (F) Violin plot showing Mitf expression in mature and proliferative states across different scRNA-seq data sets: human epidermal melanocytes (top), day 7 of the progressive pigmentation model (middle) and days 0, 3, and 5 of the progressive pigmentation model. All numerical data are listed in S1 Data. (DOCX) [file pbio.3002776.s008.docx]

**Supporting Information for**

**Distinct melanocyte subpopulations defined by stochastic expression of proliferation or maturation programs enable a rapid and sustainable Pigmentation response**

Ayush Aggarwal^1,2^, Ayesha Nasreen^1,2^, Babita Sharma^1,2^, Sarthak Sahoo^3^, Keerthic Aswin^1,2^, Mohammed Faruq^1,2^, Rajesh Pandey^1,2^, Mohit K Jolly^3^, Abhyudai Singh^4,5^, Rajesh S Gokhale^6,7^ and Vivek T Natarajan^1,2*^

Vivek T Natarajan, PhD

CSIR-Institute of Genomics and Integrative Biology

Mathura Road, Delhi 110 020, India

Phone No. 91-011-29879203

**Email:**  [tnvivek@igib.in,](mailto:tnvivek@igib.in,) tnvivek@igib.res.in


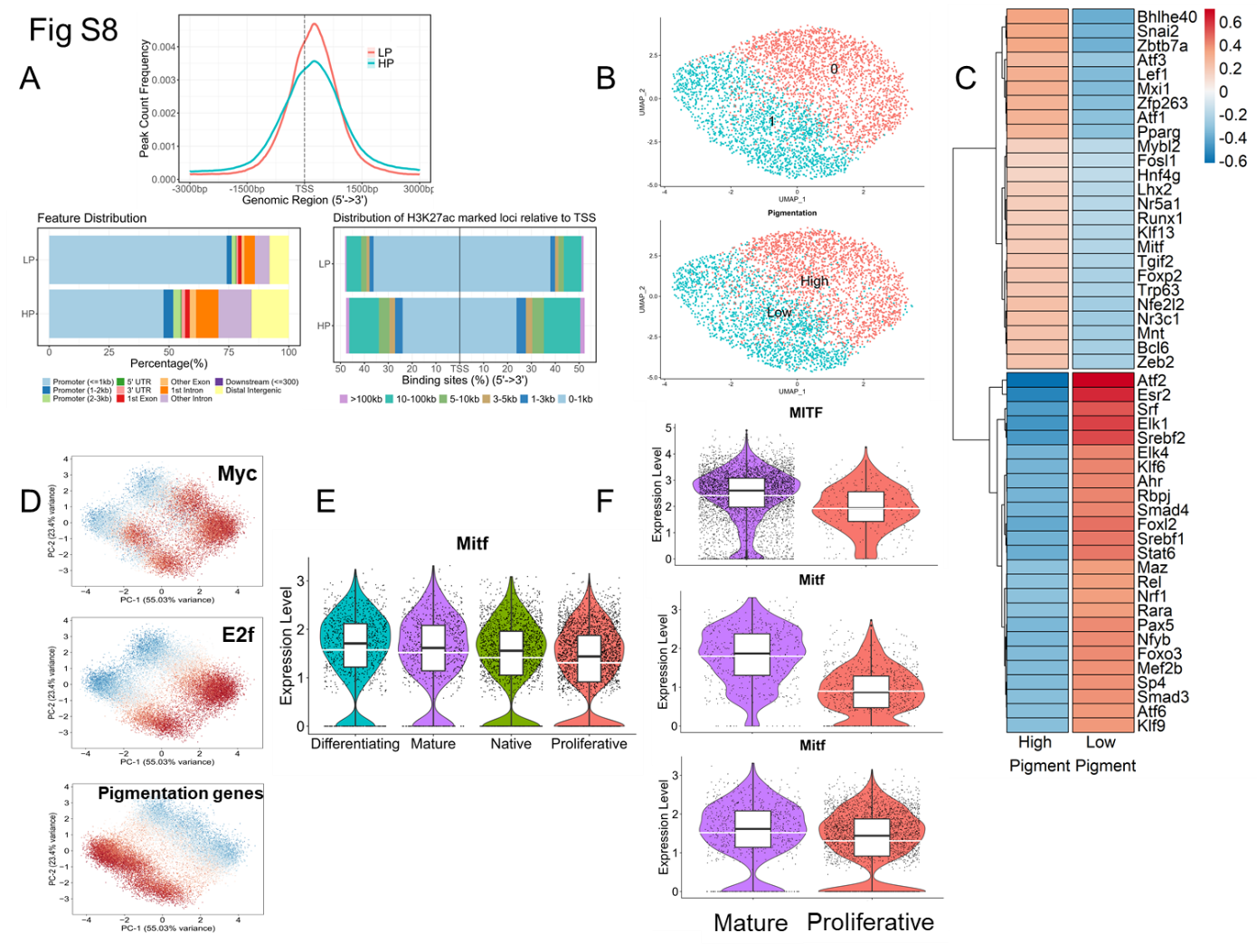


Fig S8: Analysis of H3K27ac ChIP and scRNA seq datasets (Related to Fig 4 and 5)

1. Plots showing distribution of H3K27ac peaks around the TSS (top), annotation of peaks (bottom left) and distribution of peaks relative to TSS in day5 LP and HP population.
2. UMAP plot showing day7 cells of the progressive pigmentation model colored by clusters identified using TF activity (top) and pigmentation (bottom).
3. Heatmap showing top TF active in LP and HP population at day7 (right). TF activity derived from scRNA data using Dorothea and Viper packages.
4. PCA plot showing expression of Myc (top), E2f (middle) and pigmentation genes (bottom) in the artificial cells simulated using RACIPE.
5. Violin plot showing Mitf expression in differentiating, mature, native and proliferative melanocyte states in the progressive pigmentation model.
6. Violin plot showing Mitf expression in mature and proliferative states across different scRNA seq datasets: human epidermal melanocytes (top), day7 of the progressive pigmentation model (middle) and day0, 3, 5 of the progressive pigmentation model.

All numerical data are listed in S1 data.
