# Supplementary material for: Distinct melanocyte subpopulations defined by stochastic expression of proliferation or maturation programs enable a rapid and sustainable pigmentation response
Source: PLoS Biol. 2024 Aug 20;22(8):e3002776. doi: 10.1371/journal.pbio.3002776 (PMC11364419; doi:10.1371/journal.pbio.3002776)
Supplement: S1 Raw Images — (PDF) [file pbio.3002776.s012.pdf]

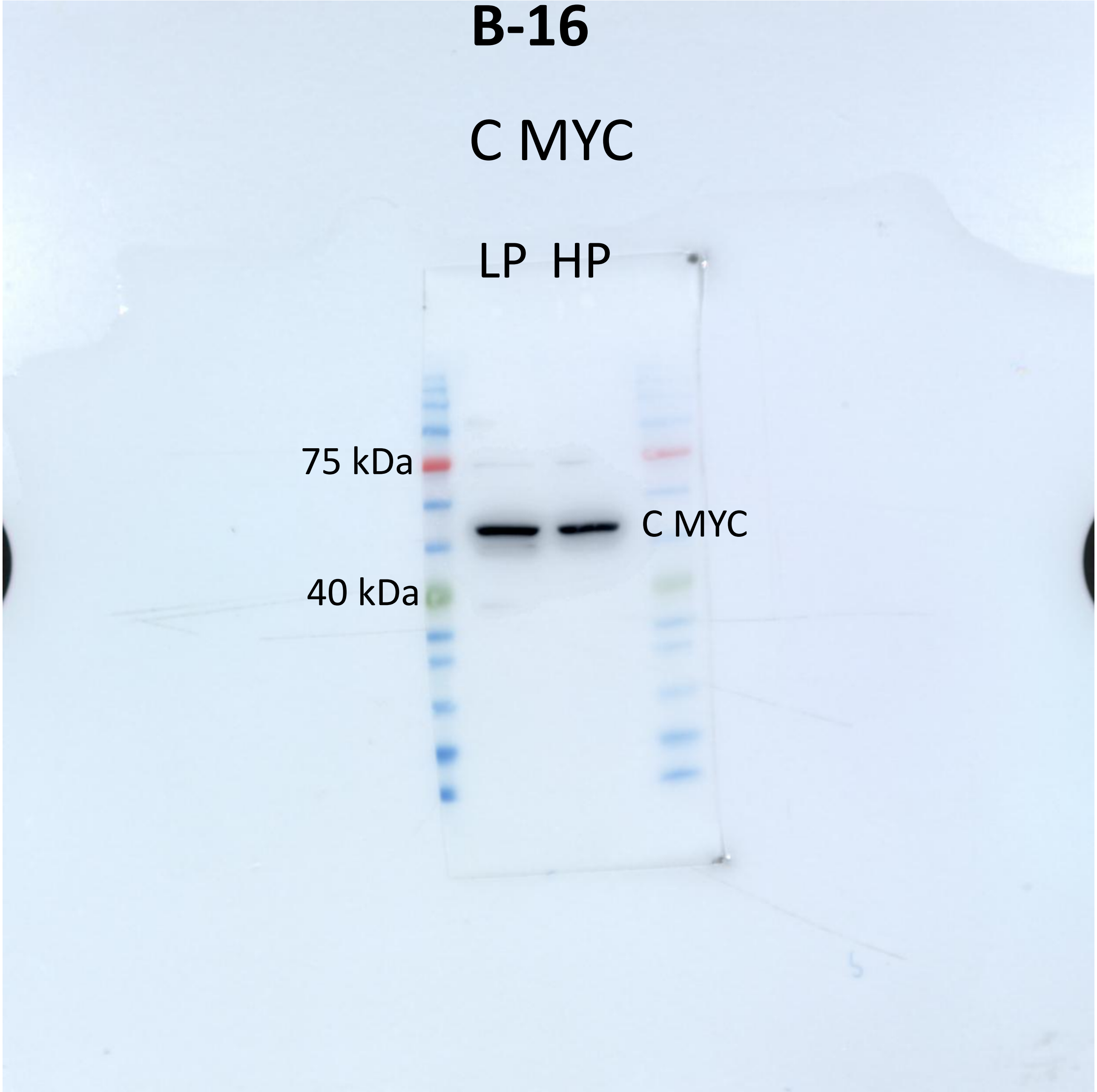

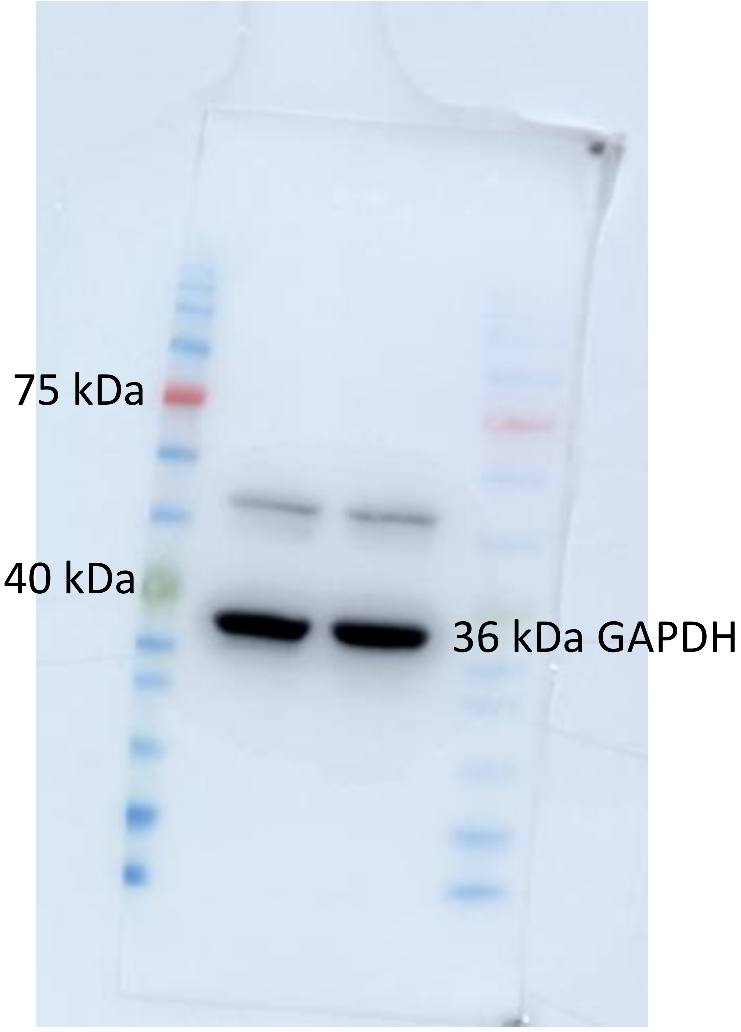

These images were used to make figure panel S6 C

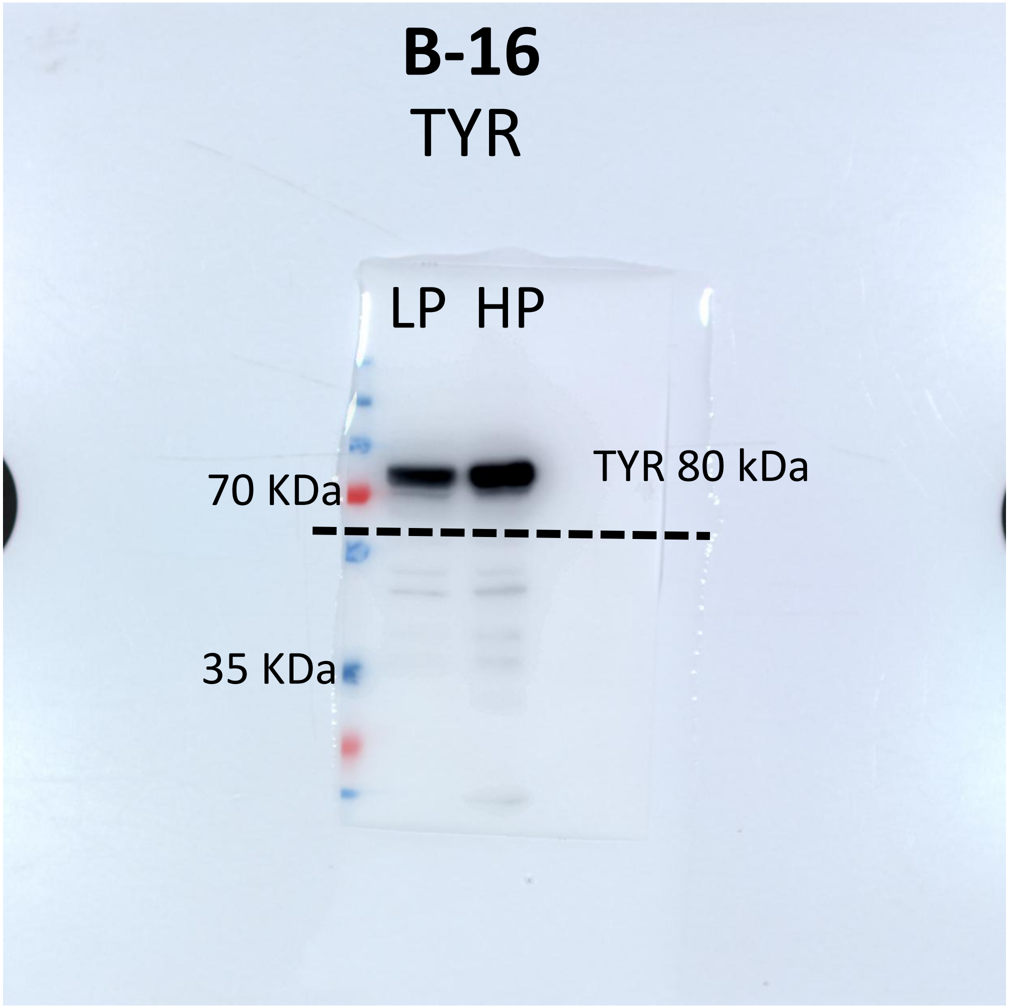

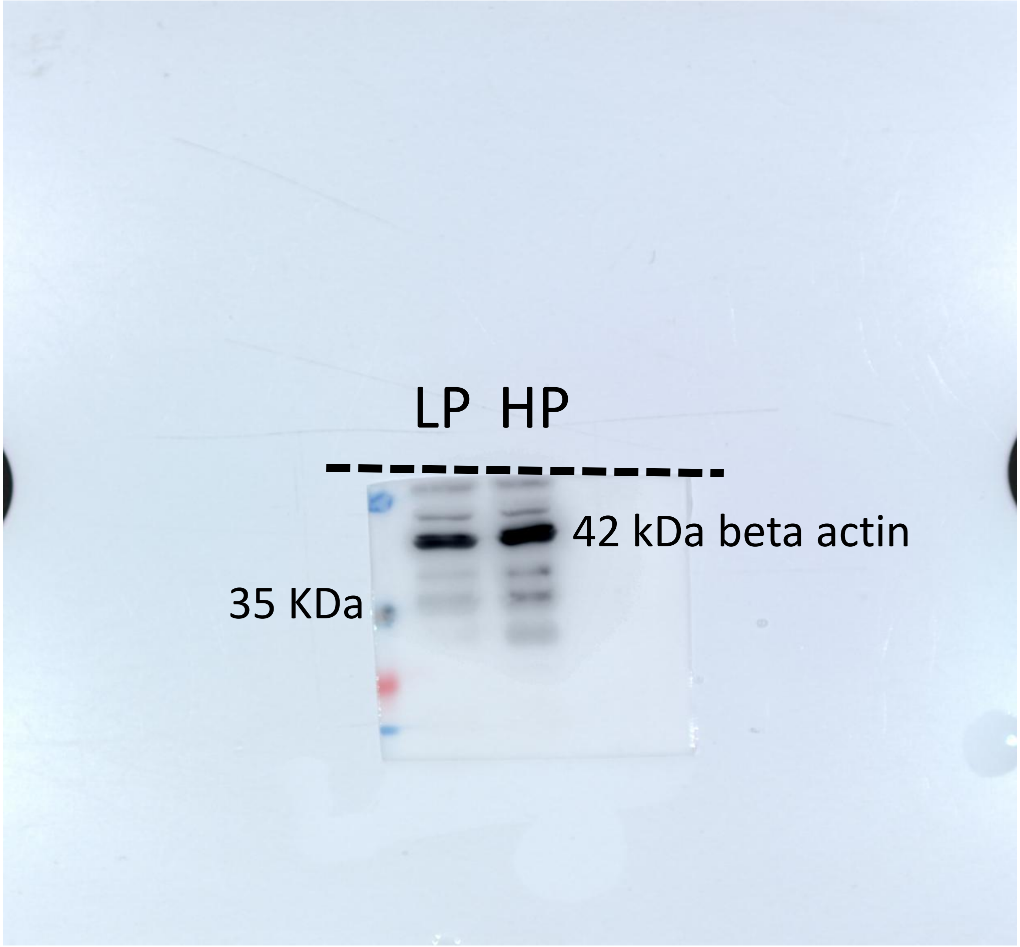

These images were used to make figure panel S6 C

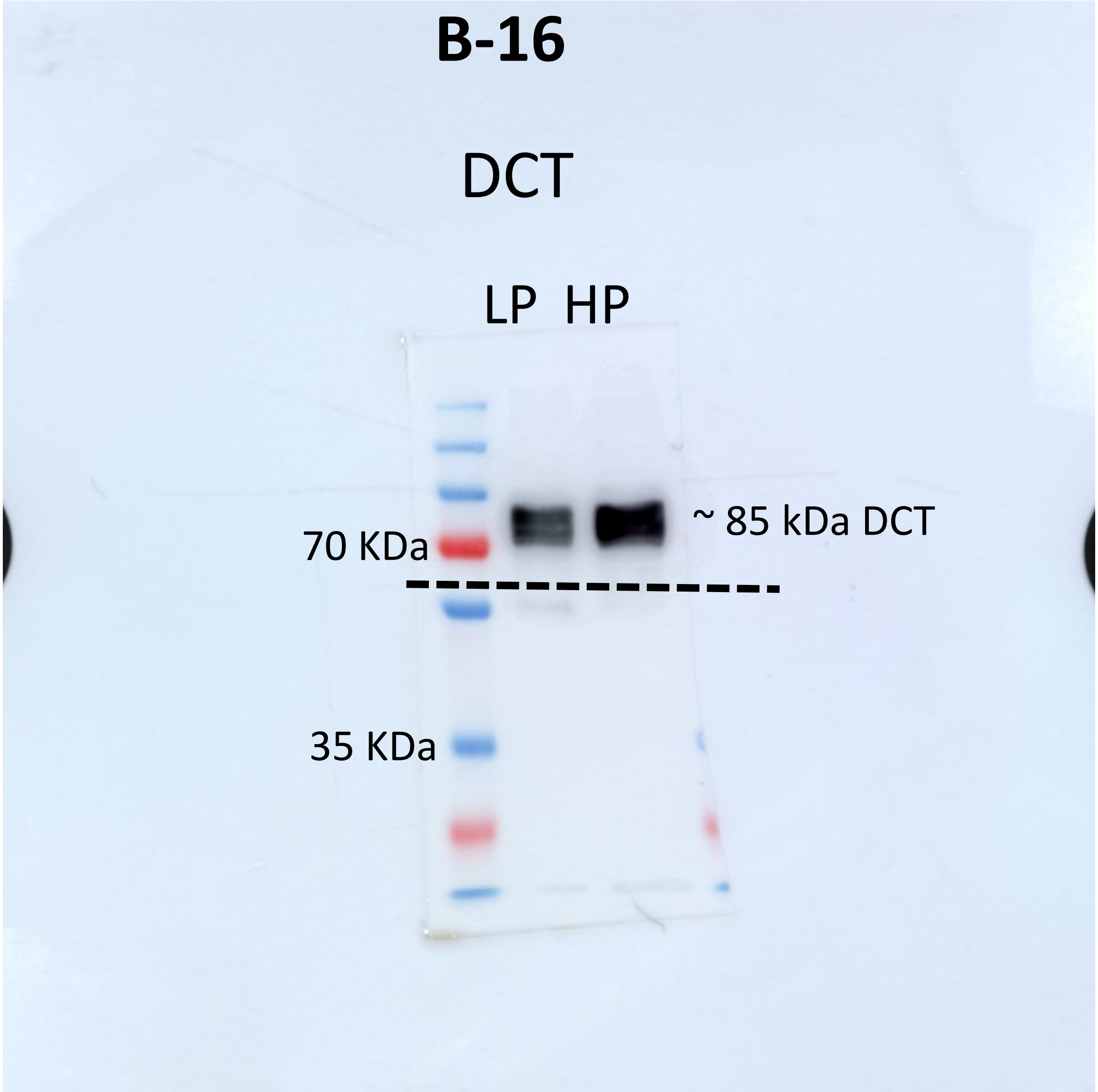

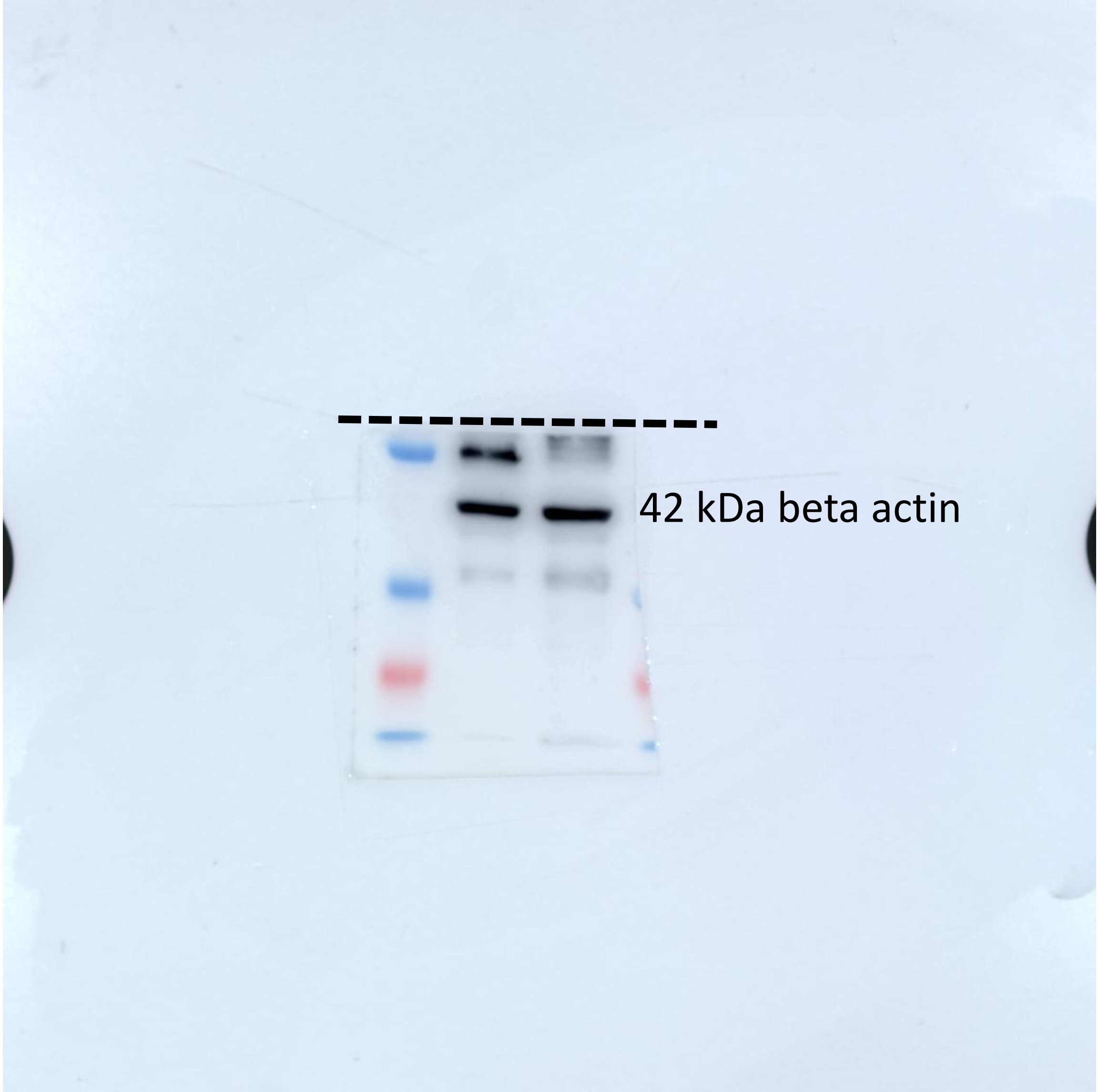

These images were used to make figure panel S6 C

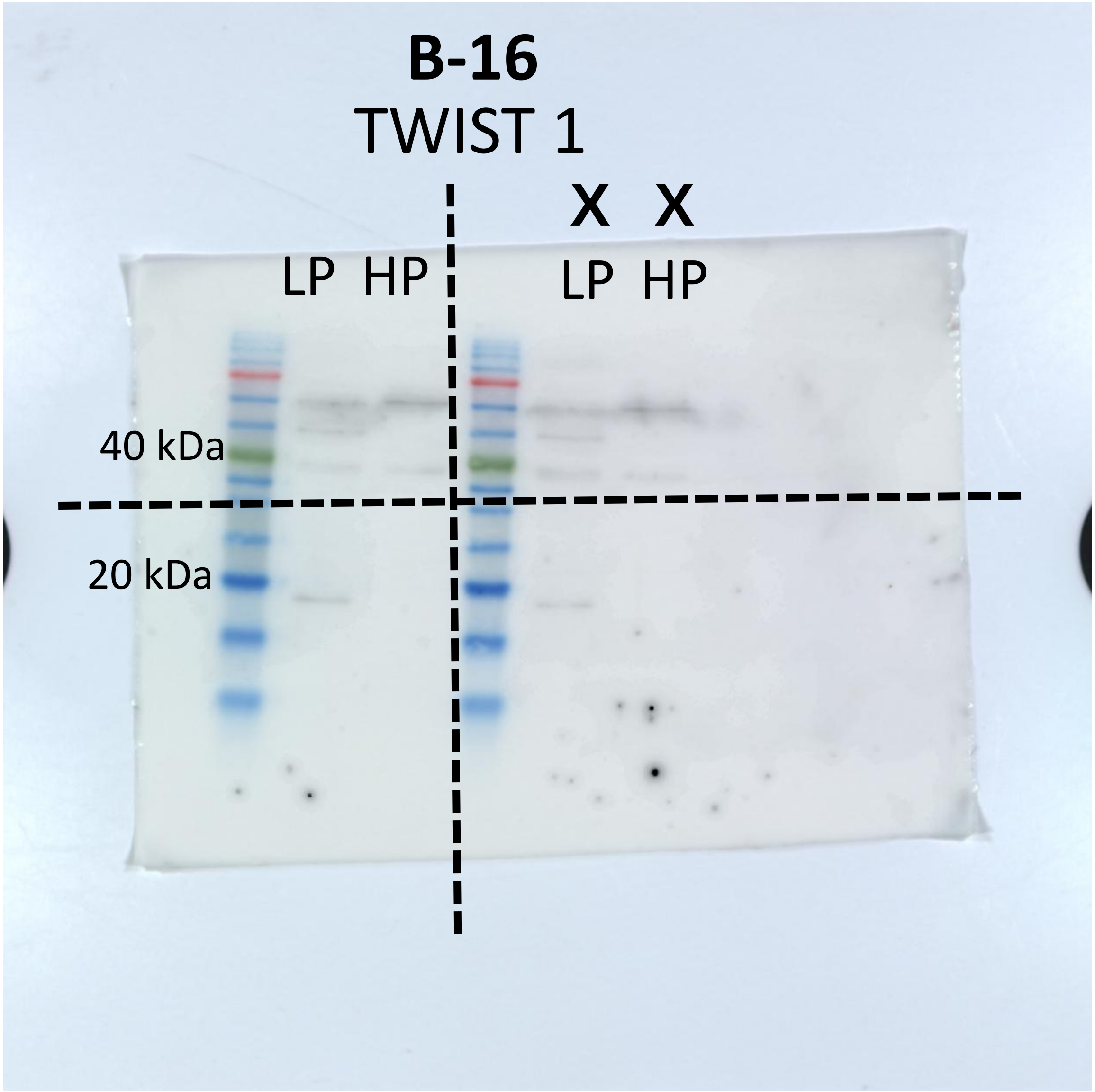

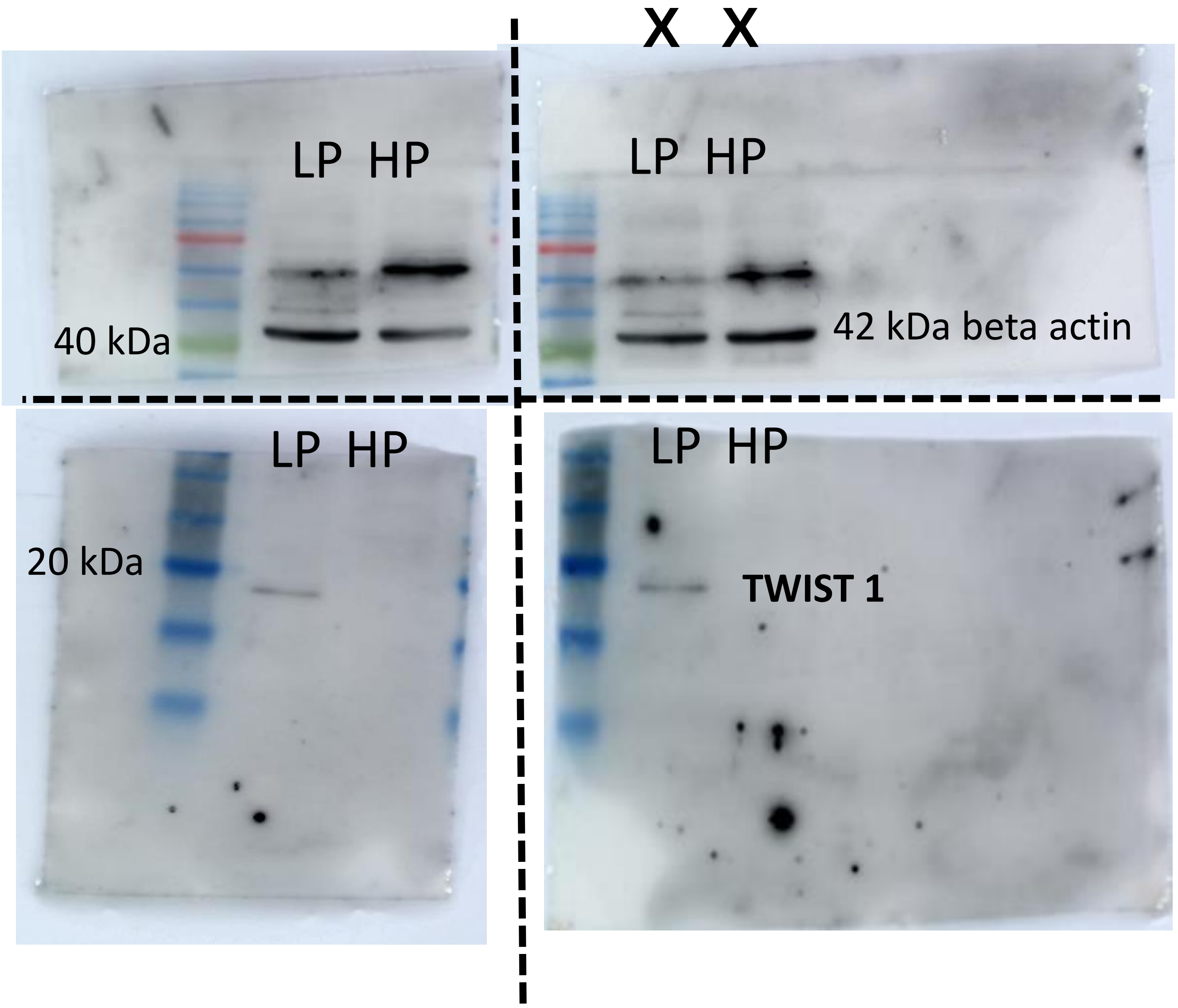

These images were used to make figure panel S6 C

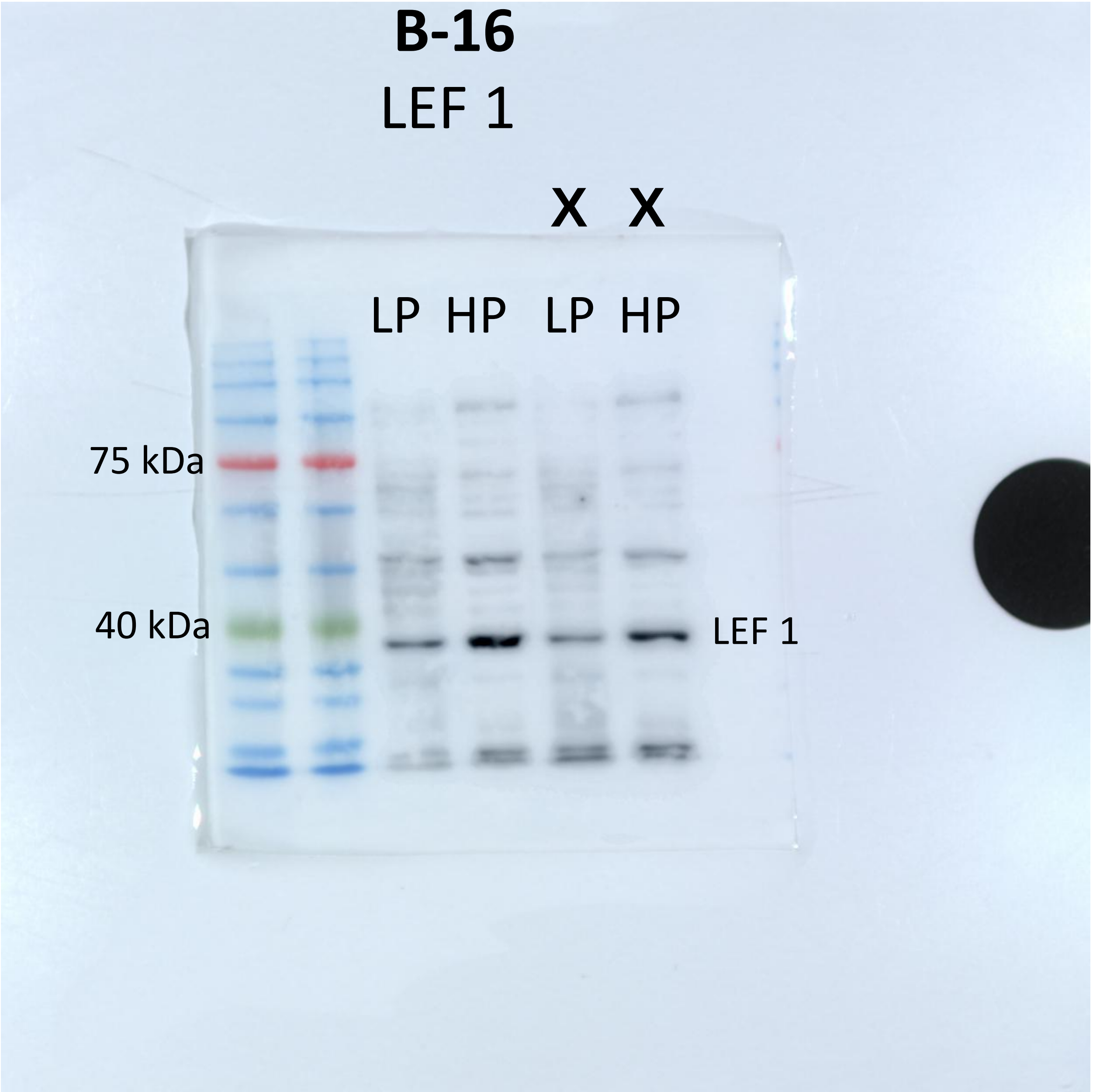

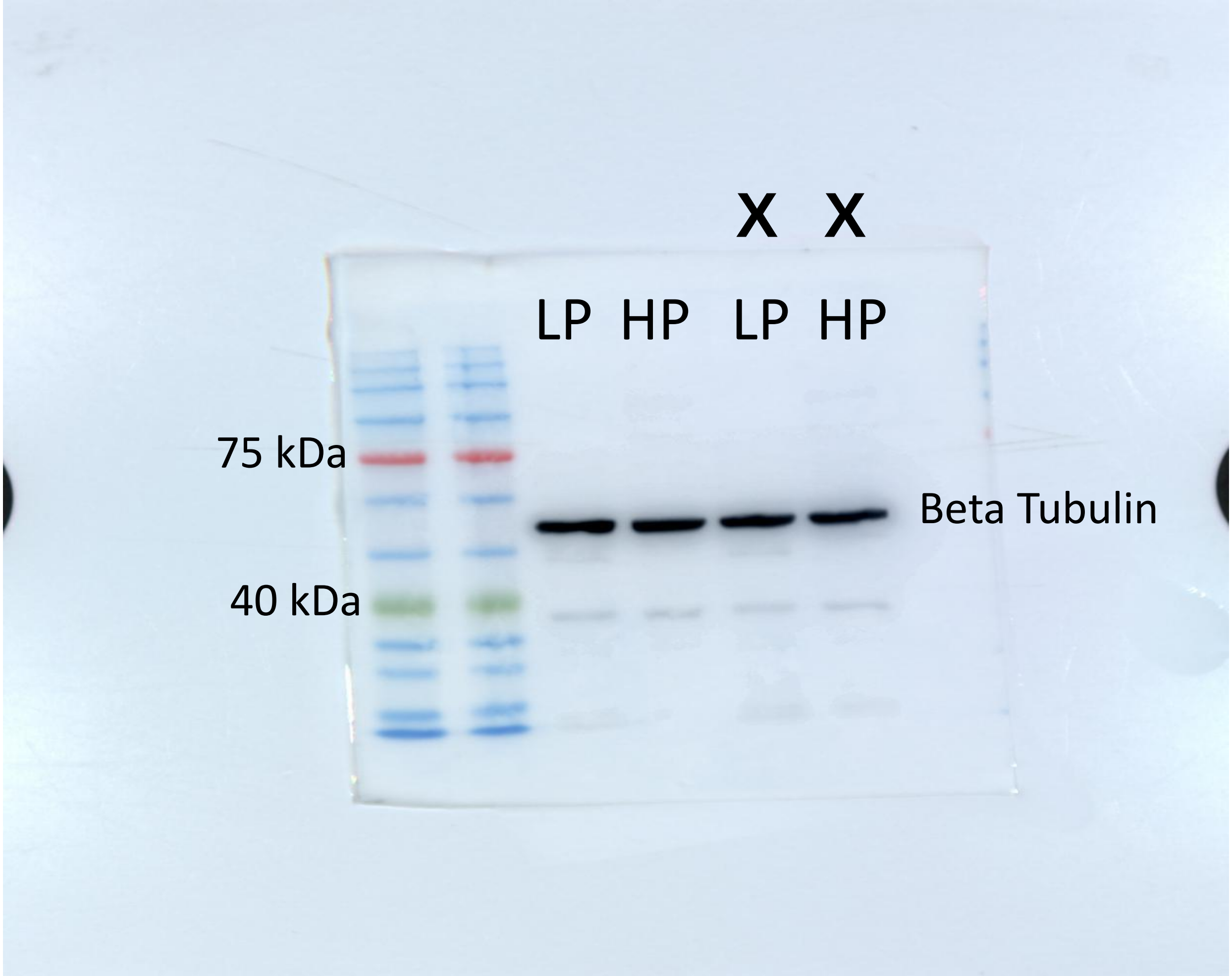

These images were used to make figure panel S6 C

MNT-1

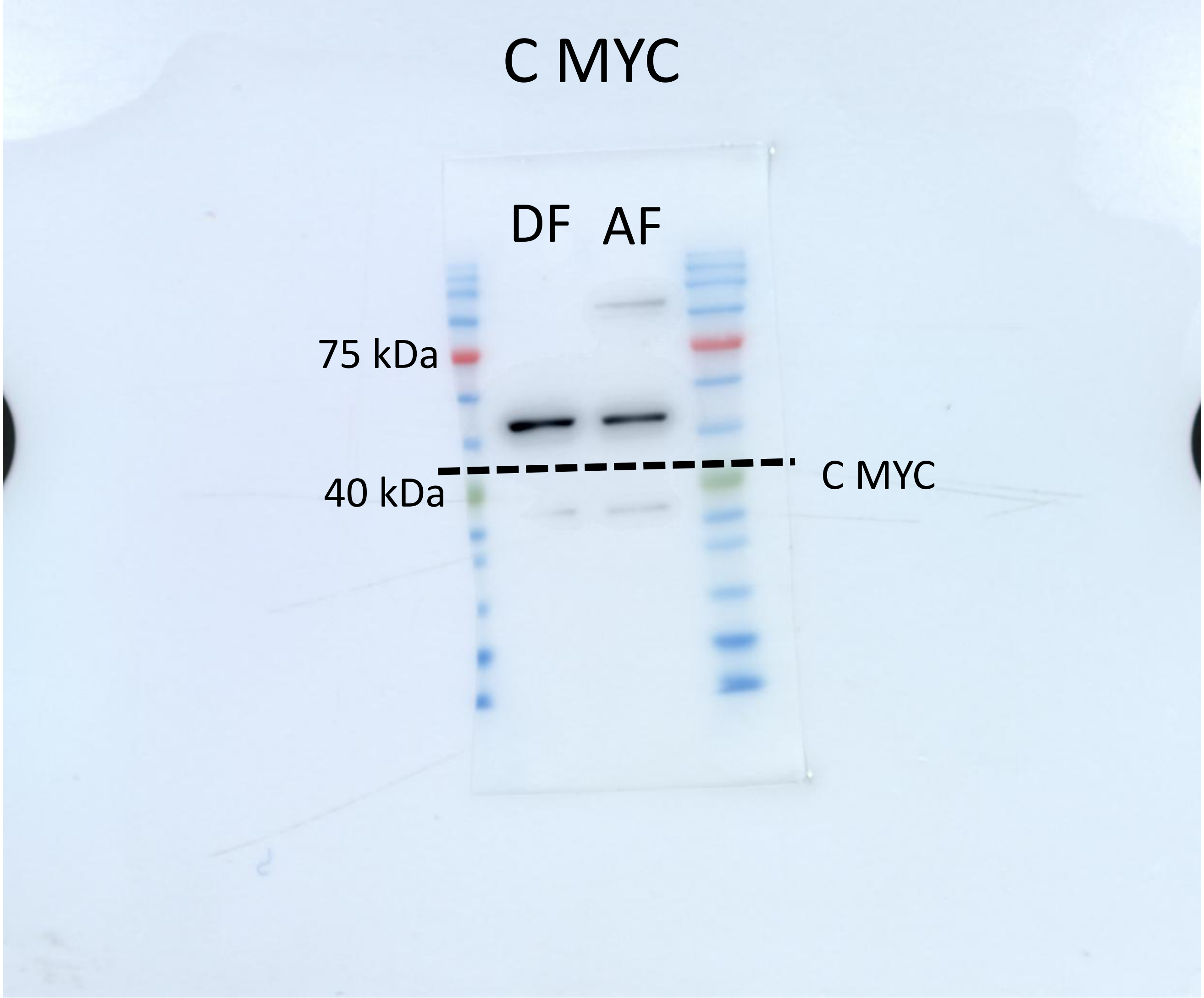

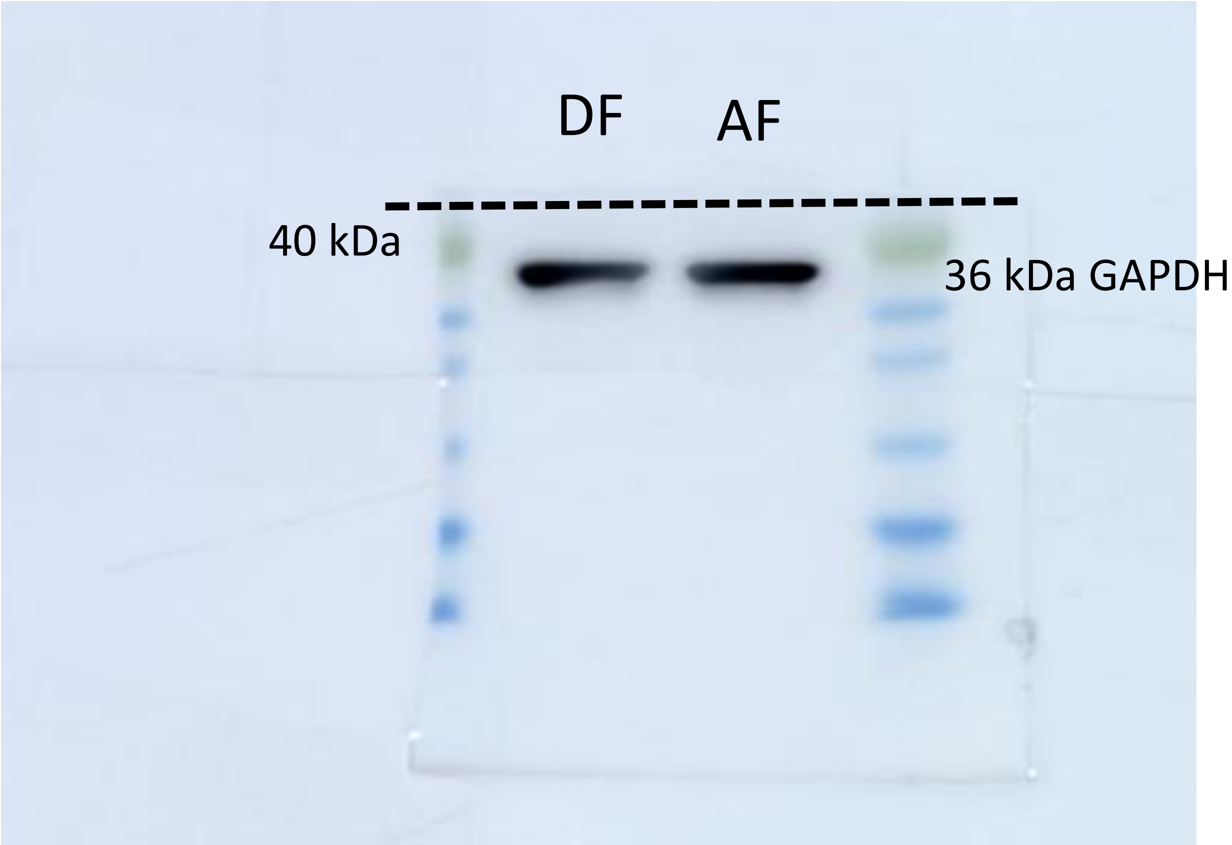

These images were used to make figure panel S4 B

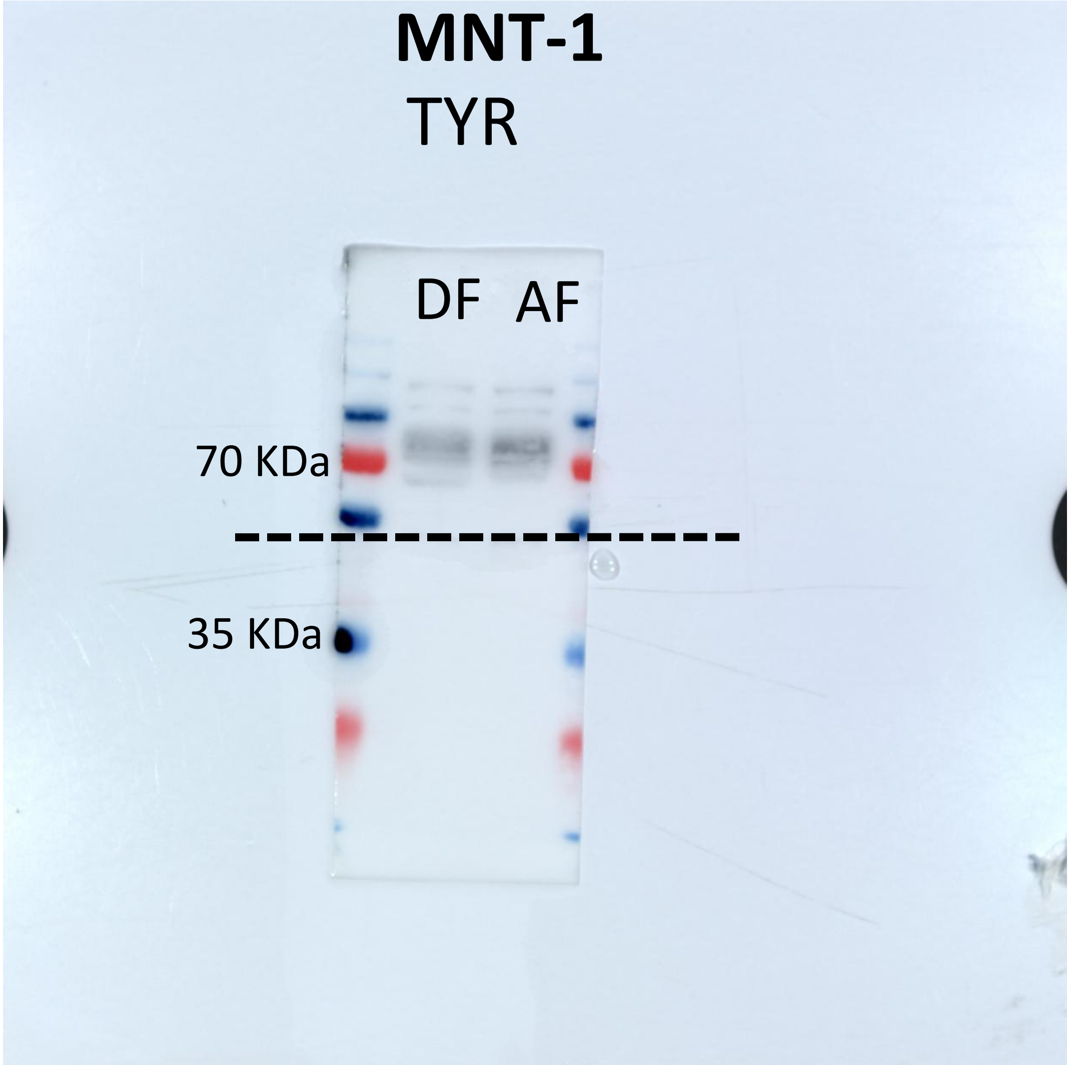

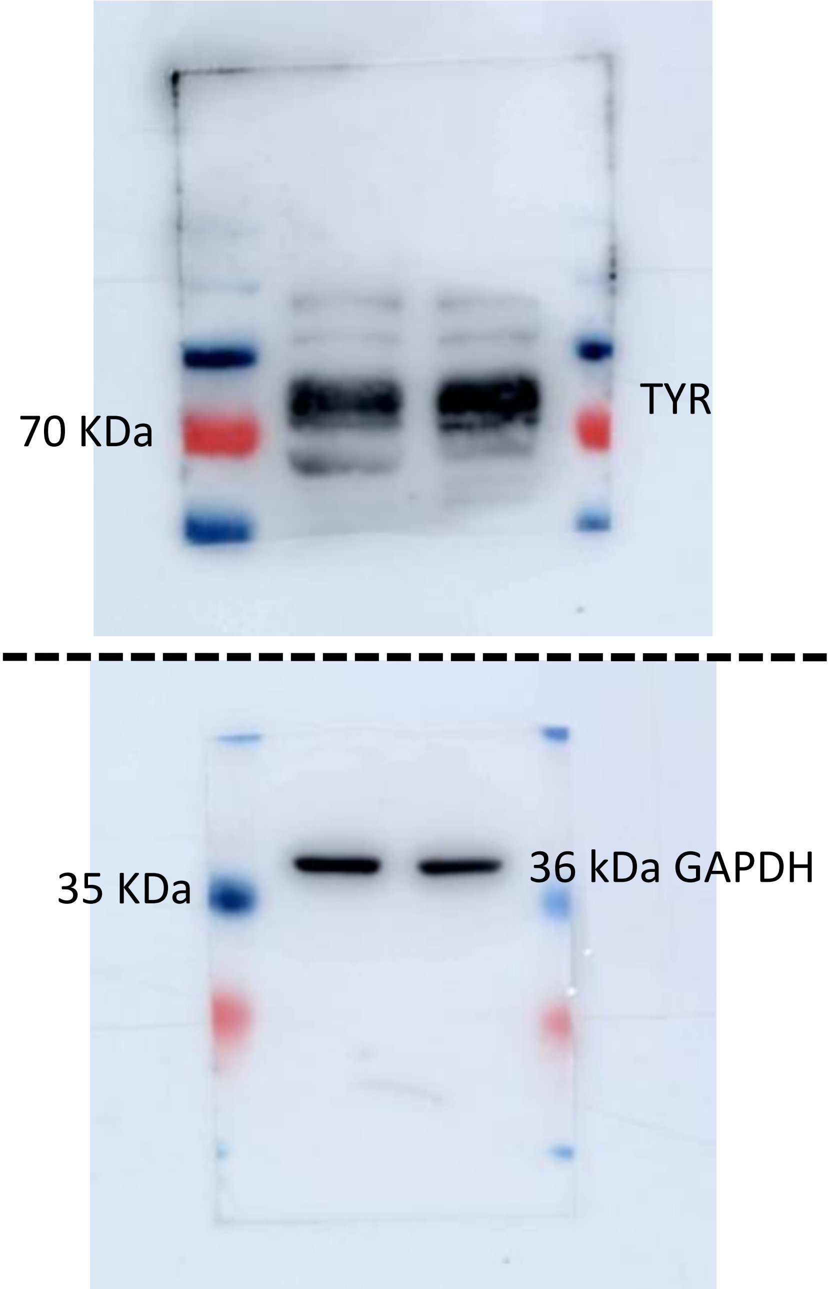

These images were used to make figure panel S4 B

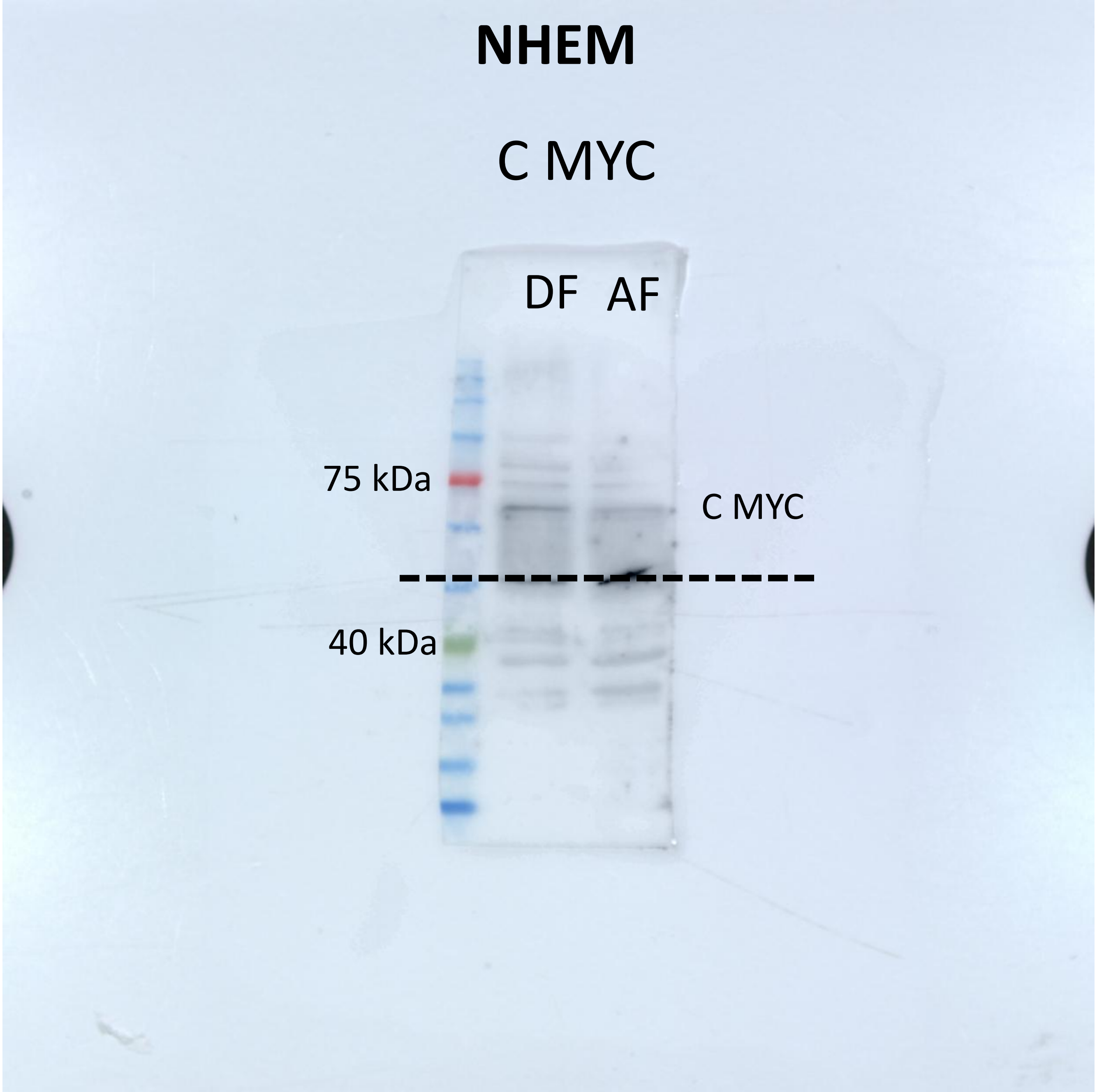

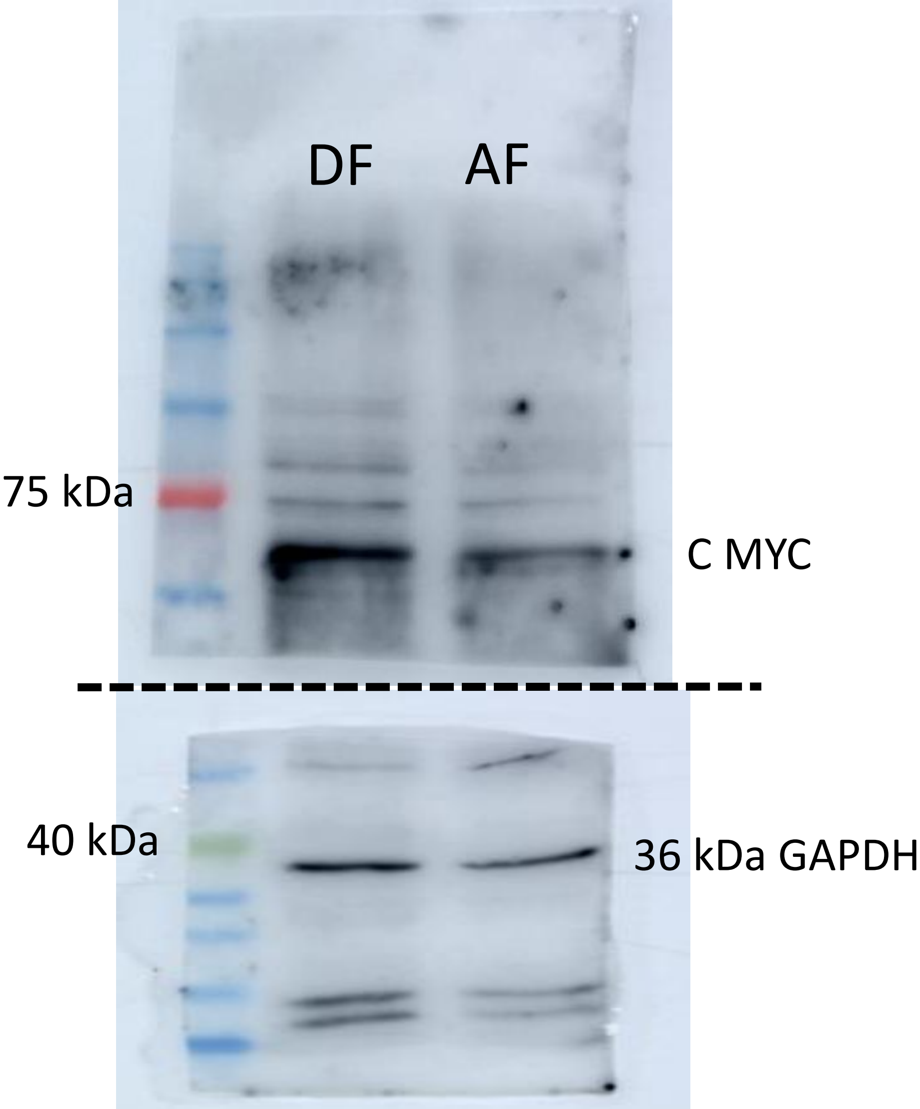

These images were used to make figure panel S3 C

NHEM

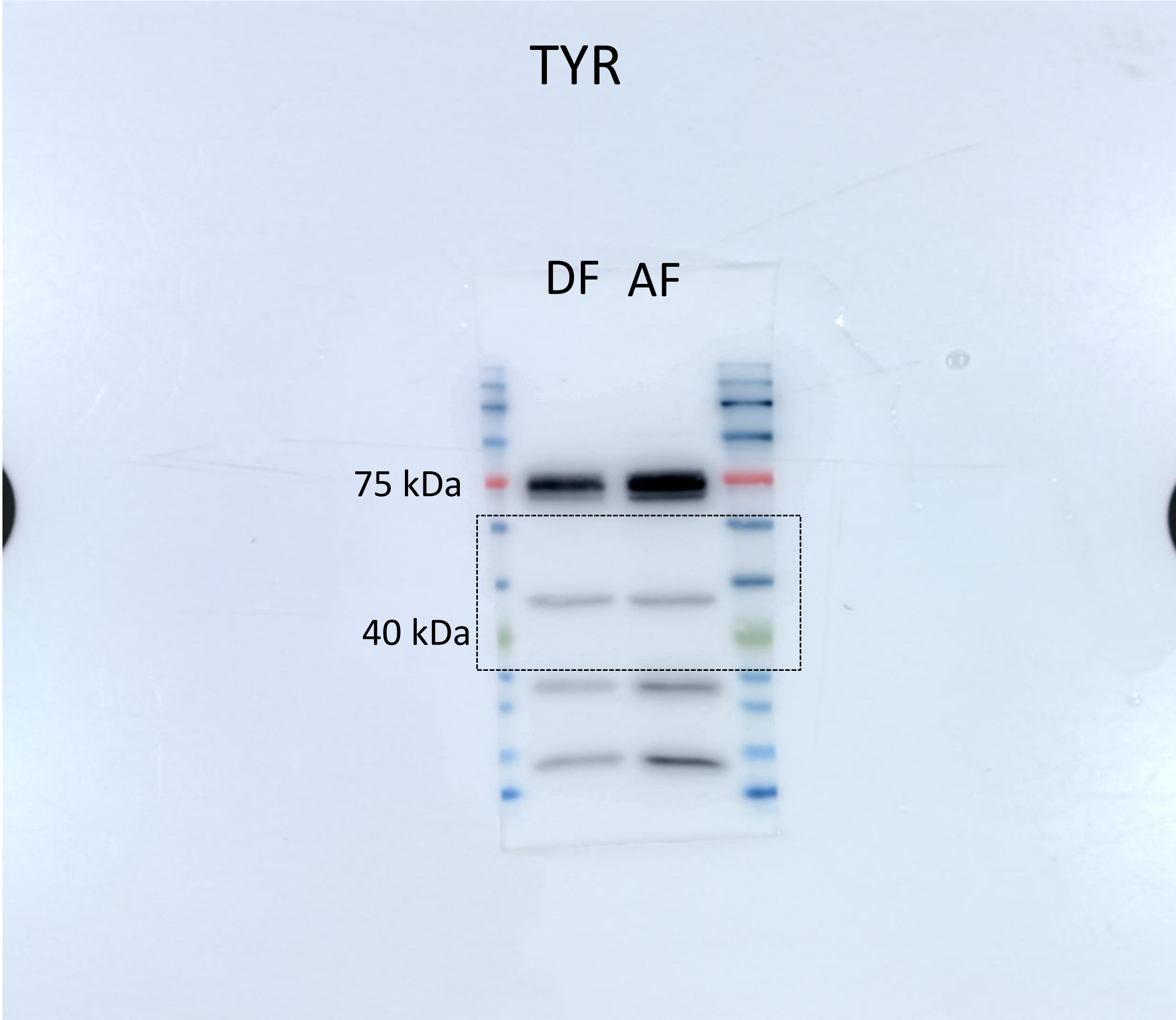

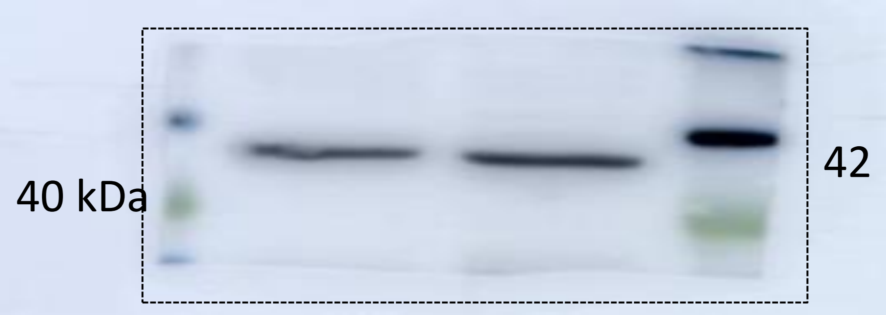

These images were used to make figure panel S3 C
