# Supplementary material for: Distinct melanocyte subpopulations defined by stochastic expression of proliferation or maturation programs enable a rapid and sustainable pigmentation response
Source: PLoS Biol. 2024 Aug 20;22(8):e3002776. doi: 10.1371/journal.pbio.3002776 (PMC11364419; doi:10.1371/journal.pbio.3002776)
Supplement: Attachment — Submitted filename: point by point response.docx [file pbio.3002776.s013.docx]

Dear Dr Natarajan,

Thank you for your patience while your manuscript entitled "Stochastic segregation of proliferation and maturation programs into distinct subpopulations enables rapid and sustainable pigmentation response" was peer-reviewed at PLOS Biology. Please also accept my sincere apologies for the delay in providing you with our decision. The manuscript has now been evaluated by the PLOS Biology editors, an Academic Editor with relevant expertise, and by two independent reviewers.

The reviews are attached below. As you will see, the reviewers find the conclusions interesting and significant for the field, however they also raise several concerns that would need to be addressed before we can consider the manuscript for publication. Reviewer 1 mentions that the manuscript should be clearer regarding novelty and general implications of the conclusions. Reviewer 2 is more critical and thinks that the cellular subpopulations have been only identified based on computational analysis of scRNA-seq data, and that independent confirmation at the protein level is needed to confirm the conclusions. This reviewer also thinks that the figures and figure legends should be improved so the readers can understand the content without having to look back at the text.

In light of the reviews, we would like to invite you to revise the work to thoroughly address the reviewers' reports. Given the extent of the revision needed, we cannot make a decision about publication until we have seen the revised manuscript and your  response to the reviewers' comments. Your revised manuscript is likely to be sent for further evaluation by all or a subset of the reviewers.

We expect to receive your revised manuscript within 3 months. Please email us ([plosbiology@plos.org](mailto:plosbiology@plos.org)) if you have any questions or concerns, or would like to request an extension.

At this stage, your manuscript remains formally under active consideration at our journal; please notify us by email if you do not intend to submit a revision so that we may withdraw it.

**Our Response:** We express our heartfelt gratitude to the Editorial team for their invaluable support throughout this process. Following meticulous consideration of the reviewers' comments, we have found them to be constructive, greatly aiding us in articulating our arguments effectively. Subsequently, we have undertaken a comprehensive series of validating experiments, including western blot analysis and immunofluorescence experiments, often incorporating FACS to sort cells based on melanin content. These new findings provide strong support for the conclusions drawn from single-cell data. Building upon this, we have significantly revised both the text and figures of the manuscript. Additionally, we have addressed the comment regarding the descriptions in Figure legends, ensuring they are accurately represented in this revised version. We sincerely believe that these revisions enhance the manuscript's suitability for publication in PLoS Biology.

**IMPORTANT - SUBMITTING YOUR REVISION**

Your revisions should address the specific points made by each reviewer. Please submit the following files along with your revised manuscript:

1. A 'Response to Reviewers' file - this should detail your responses to the editorial requests, present a point-by-point response to all of the reviewers' comments, and indicate the changes made to the manuscript.
*NOTE: In your point-by-point response to the reviewers, please provide the full context of each review. Do not selectively quote paragraphs or sentences to reply to. The entire set of reviewer comments should be present in full and each specific point should be responded to individually, point by point.
You should also cite any additional relevant literature that has been published since the original submission and mention any additional citations in your response.

2. In addition to a clean copy of the manuscript, please also upload a 'track-changes' version of your manuscript that specifies the edits made. This should be uploaded as a "Revised Article with Changes Highlighted" file type.

3. Resubmission Checklist
When you are ready to resubmit your revised manuscript, please refer to this resubmission checklist: <https://plos.io/Biology_Checklist>

To submit a revised version of your manuscript, please go to <https://www.editorialmanager.com/pbiology/> and log in as an Author. Click the link labelled 'Submissions Needing Revision' where you will find your submission record.

Please make sure to read the following important policies and guidelines while preparing your revision and fulfil the editorial requests:

a) *PLOS Data Policy*
Please note that as a condition of publication PLOS' data policy (<http://journals.plos.org/plosbiology/s/data-availability>) requires that you make available all data used to draw the conclusions arrived at in your manuscript. If you have not already done so, you must include any data used in your manuscript either in appropriate repositories, within the body of the manuscript, or as supporting information (N.B. this includes any numerical values that were used to generate graphs, histograms etc.). Please also indicate in each figure legend where the data can be found. For an example see here: <http://www.plosbiology.org/article/info%3Adoi%2F10.1371%2Fjournal.pbio.1001908#s5>

b) *Published Peer Review*
Please note while forming your response, if your article is accepted, you may have the opportunity to make the peer review history publicly available. The record will include editor decision letters (with reviews) and your responses to reviewer comments. If eligible, we will contact you to opt in or out. Please see here for more details:
<https://blogs.plos.org/plos/2019/05/plos-journals-now-open-for-published-peer-review/>

c) *Blot and Gel Data Policy*
Please provide the original, uncropped and minimally adjusted images supporting all blot and gel results reported in an article's figures or Supporting Information files. We will require these files before a manuscript can be accepted so please prepare them now, if you have not already uploaded them. Please carefully read our guidelines for how to prepare and upload this data: <https://journals.plos.org/plosbiology/s/figures#loc-blot-and-gel-reporting-requirements>

d) *Blurb*
Please also provide a blurb which (if accepted) will be included in our weekly and monthly Electronic Table of Contents, sent out to readers of PLOS Biology, and may be used to promote your article in social media. The blurb should be about 30-40 words long and is subject to editorial changes. It should, without exaggeration, entice people to read your manuscript. It should not be redundant with the title and should not contain acronyms or abbreviations. For examples, view our author guidelines: <https://journals.plos.org/plosbiology/s/revising-your-manuscript#loc-blurb>

e) *Protocols deposition*
To enhance the reproducibility of your results, we recommend that if applicable you deposit your laboratory protocols in [protocols.io](http://protocols.io/), where a protocol can be assigned its own identifier (DOI) such that it can be cited independently in the future. For instructions see: <https://journals.plos.org/plosbiology/s/submission-guidelines#loc-materials-and-methods>


Thank you again for your submission to our journal. We hope that our editorial process has been constructive thus far, and we welcome your feedback at any time. Please don't hesitate to contact us if you have any questions or comments.

Sincerely,

Ines
--
Ines Alvarez-Garcia, PhD
Senior Editor
PLOS Biology
[ialvarez-garcia@plos.org](mailto:ialvarez-garcia@plos.org)

Reviewers' comments

Rev. 1:
The authors examined melanocytes derived from multiple sources, including human, zebrafish, and mouse cell lines, using scRNA seq multi-omics. The analyses identified the pigment (mature) and proliferation states, reflecting the heterogeneity of melanocyte cell states. The analyses look overall reliable, and I had only a few questions/comments for clarification (see below). However, the novelty and implications of the conclusion could have been clearer. Was it unexpected to see the heterogeneity of the cell state among melanocytes in humans and zebrafish or in culture? If it was an expected finding, what would this result add to the advancement of the field? Clarifying these will be helpful for the general readers.

**Our Response:** We thank the reviewer for finding our analyses reliable and point us to make our conclusions available to a broader audience. The anticipated variability of cell states in Melanocytes derived in situ, including Human epidermal melanocytes directly sorted from skin and Zebrafish melanocytes, may be attributed to microenvironmental differences. However, we did not expect the extent of heterogeneity in primary cultured melanocytes as well clonal B16 cells, prior to performing the scRNA seq. Recent findings from scRNA seq platforms indicate that intra-population heterogeneity in cultured cancer cell-lines primarily stems from the presence of extrachromosomal DNA and copy number variations, with epigenetic factors playing additional role (Zhu et al., Nat Comm, December 2023). Further, barcoding-based tracking revealed that the daughter cells from the same progenitor might beget different transcriptomic status. Perturbation such as hypoxia results in division of one cluster into many, indicating that the cells within the cluster respond differently to the same perturbation.

We gather that this aspect was framed with insufficient clarity and in the revised manuscript we have elaborated these points to bring in the necessary changes to make this point clear.

The authors convey the melanocyte response to UV radiation in the introduction. However, it needs to be clear whether the cell states they analyze are related to UV response or homeostasis. What percentage of melanocytes proliferate and show pigment state under homeostasis and after sun exposure? The portion of each cell state is expected to be distinct between different physiological conditions.

**Our Response:** Based on our data we demonstrate that UV (fsk treatment) does not result in some new state that emerges, but the pre-existing proliferative and pigmenting populations seen under homeostatic state is differentially impacted with this perturbation. In the scRNA seq data we did not have the untreated melanocytes to compare, hence this conclusion was not very apparent. But now with ICC and FACS based monitoring we are able make this point clear. Ki67 +ve cells reduce from around 50% to 30% between frequent and infrequent UV exposure. The Tyr positive cells increase from 88% to 96%.

Figure 1-5: Can the authors show representative pictures for high- or low-pigmentation melanocytes?

**Our Response:** We have now included representative images of individual cells as well as colonies that have high or low pigmentation in the supplementary Fig S5 and 6.

Figure 1E: The result description does not correspond to the figure.
Figure 5F, G should be labelled correctly.

**Our Response:** Thank you for pointing this out, our apologies for the mistake. This has now been modified.

Rev. 2:
This is a manuscript entitled by Aggarwal et al. entitled "Stochastic segregation of proliferation and maturation programs into distinct subpopulations enables rapid and sustainable pigmentation response". In this manuscript, the authors address the question of whether a defined population of melanocytes may occupy more than one differentiation state. They propose that such heterogeneity might, for example, account for the distinct responses of proliferation and differentiation exhibited by melanocytes upon exposure to UV irradiation. To attempt to answer this question, they analyze multiple sets of single-cell expression data to explore melanocyte cell heterogeneity across species, conditions, and transformation state to define distinct population states. With this information, they develop a gene regulatory network (GRN) model which links the pigmentation transcription factor genes MITF and LEF1 and which they use to generate computationally the existence of distinct,
steady-state differentiation states that are observed experimentally in their scRNA-Seq analyses.

The datasets used and acquired for this manuscript are as follows:
(1) Reanalysis of scRNA data from normal human epidermal melanocytes (NHEM) obtained from a previous melanocyte study and published in 2021 (Belote et al., GSE151091);
(2) EGAS00001002927, whose use is described on page 5 and in Figures S2A-C (but otherwise not referenced);
(3) Melanocytes from zebrafish embryos at 24 hpf which were analyzed by scRNA-Seq;
(4) Forskolin daily- and alternate daily-pulsed NHEMs to drive proliferation or pigmentation respectively per Malcov-Brog et al., 2018; scRNA-Seq analysis;
(5) scRNA-Seq analysis of murine B16 melanoma cells separated into low pigmentation and high pigmentation phenotypes (Figure 2A-2D); also used for trajectory analysis (Figure 2E,F)
(6) Additional correlation of mouse hair follicle scRNA-Seq data from Infarinato et al. (2020) (Fig. S3E)
(7) scMulti-omics (RNA- and ATAC-seq) sequencing of cells in the progressive pigmentation model (B16) at days 0, 3, and 5, determining differentially accessible regions (DARs);
(8) H3K27Ac ChIP-Seq of B16 cells at day 5 of the pigmentation model, determining differentially and
The DAR and DHAcR data was used to enhance an additional analysis of (5) above to infer the transcription factors that are active in the low pigmentation (LP) and high pigmentation (HP) states.


Some general comments are as follows:

(1) The fundamental weakness of the manuscript in its current form is a lack of rigor and lack of independent validation (i.e. insufficient validation) of the characterizations of cellular subpopulations that are described after the experimental perturbations described above. This is especially true for (4) (same- and alternate-day fsk-pulsed NHEMs) and (5) (scRNA-Seq analysis of B16 melanoma cells). The authors have not independently validated the existence of distinct cell subpopulations at the cellular level by examining individual cells for their expression or non-expression of the specific markers which can distinguish the subpopulations they claim are revealed by the scRNA data and subsequent analytical dimensionality reduction.

**Our Response:** We thank the reviewer for pointing out to the critical validation experiments. We have now performed the Western blot, ICC and FACS based analysis which substantiates the claim made earlier solely based on the scRNA seq analysis. Our validation experiments indicate that indeed the changes observed at the RNA level are true when the protein levels of segregated population are analyzed by western blot analysis. ICC for proliferation and pigmentation, marked by Ki67 and Tyr, are more and less respectively in the FACS sorted low and high pigmenting B16 cells. In daily and alternate day treated NHEMs as well as MNT1 cells we demonstrate that the mean total fluorescence intensity of Ki67 and Tyr follow the expected pattern. Furthermore, using FACS based analysis we demonstrate that the proportion of Ki67 +ve cells decrease and Tyr +ve cells increase with alternate day fsk treatment.

Although this validation could be performed in different ways, one method would involve using markers that might distinguish the subpopulations (i.e. MITF or TYR, MKI67, and TWIST1) and immunostaining cells to score them for non-overlapping,
high level expression of these markers. For example, to validate the data, one would expect that the proportions of MITF+/MKI67-/TWIST1-, MITF-/MKI67+/TWIST1-, and MITF-/MKI67-/TWIST1+ cells in a population, scored for expression after immunofluorescence, should correspond to the proportions of those populations described in the scRNA-Seq analysis. The important conclusions of this manuscript are predicated upon the existence of these cellular subpopulations; however, in the current version of the manuscript, they are only demonstrated on the basis of computational analysis of scRNA-Seq data. Since the interpretation of scRNA-Seq is subject to the application of discretionary and what can be arbitrary parameters and constraints applied to the high-dimensional data, in my opinion independent confirmation at the protein expression level of these subpopulations is necessary to accept the conclusions of the manuscript as valid.

**Our Response:** Thank you for providing further detailed comments on this point. As mentioned earlier, we have conducted protein level investigations in sorted cells based on melanin content, along with immunofluorescence studies utilizing select markers. Specifically, in B16 cells, we have conducted double immunofluorescence analysis using MKi67 and Tyr markers. Our findings indicate that pigmenting cells exhibit high Tyr and low MKi67 levels, while proliferating cells display low pigment content, low Tyr, and high MKi67 levels. This observation reinforces the presence of independent states coexisting within the cell population.

(2) In my opinion, the figures and figure legends are incompletely annotated. It should be possible at a minimum to appreciate the overall content of a figure and its legend simply from viewing the figure and the legend together, without constant reference to the manuscript text. However, that is not possible with this manuscript. There are too many specific examples to describe completely, but one example is Figure 4 and its legend. It is not apparent from reviewing the figure and legend together that the data represents the results of scATAC-seq and ChIP-Seq experiments. These facts are not directly stated in the figure or the legend. I am not going to offer specific suggestions about the enhanced level of detail that needs to be provided to understand the figures and legends adequately, since this should represent common scientific writing practices, but in general the dearth of information applies to all figures and legends and they all must be improved to render the manuscript more comprehensible.

**Our Response:** Thank you for bringing this to our attention. We acknowledge and apologize for the lack of clarity in the figure legends. We have revised all figure legends to include sufficient information to understand the content of each figure. We trust that this addresses the issue appropriately.

Some specific comments follow:

1. p.3, introduction - There seems to be some unfounded assumptions in the introductory statements provided about the stress and proliferation responses. The authors state that the "stress response (which) includes proliferation, … , to prevent depletion of the melanocyte population". To impute that proliferation occurs specifically to prevent depletion of the melanocyte population appears to me to be an unsupported statement, with no direct evidence to prove that the "reason" for the stress-induced proliferation is to prevent depletion versus, for example, simply to provide a larger number of melanocytes to enhance photoprotection. Similarly, the authors imply that "the pigmentation process", through its induction of a "terminal differentiation state" in turn "reduces the likelihood of cell proliferation upon subsequent UV exposure". Again, I know of no direct evidence to support these statements. Senescence might reasonably reduce the propensity for subsequent cell
proliferation, but I am not aware of any absolute inverse relationship between "differentiation" and "likelihood of cell proliferation". The authors should strive to limit their statements and conclusions to those that can be supported by evidence.

**Our Response:** We recognize that in our discussion of the UV scenario, our suggestions may have inadvertently appeared as statements rather than inquiries. We have accordingly revised this section of the introduction and cited an earlier reference that initially addressed this dichotomy. We trust that these adjustments have brought about the necessary clarity.

2. Also p.3 - the authors refer to studies which "suggest the existence of melanocyte states other than the terminally differentiated pigmented state in the epidermis", following with "the coexistence of such transcriptionally distinct melanocyte sub-populations in the same environment is not yet known" and that their "interchange between states remains speculative". The authors seem not to have considered work described in Sun Q et al. (2023) Nature 616, 774 and Joshi et al. (2019) PLoS Genet 15(4):e1008034 that describes the existence of melanocytic cells in the same or similar environments in distinct differentiation states and their interconversion between states.

**Our Response:** We agree with the observation and have now suitably modified the text to bring in clarity. We agree that the studies referred by the reviewer talks about the existence of distinct melanocytic differentiation states and interconversion potential, the environmental cues such as UV causing such changes was not known.

3. Also p. 3 - unsupported, unreferenced statements such as "Heterogeneity within a seemingly homogeneous population is considered to provide the means to maintain the diversity required for the survival of the overall population" should be either avoided or fully substantiated by references.

**Our Response:** We have changed the statement to read “We hypothesize that the diversity inherent in a seemingly uniform population can facilitate rapid adaptation by enabling swift interchangeability when needed.”

4. P. 4 - by "generic feature' do you mean "inherent feature"?

**Our Response:** Yes. We have changed this accordingly.

5. Supplementary Figure 1 - the authors refer to the "stacked bar plot" for panel B, but to me panel B looks like a UMAP plot, not a stacked bar plot. The authors should make this correction and determine whether any similar corrections in Figure legends are required.

**Our Response:** Thank you for bringing this to our notice. Our apologies for the mistake. This has now been corrected.

6. P. 5, bottom - the authors refer to "another publicly available human dataset", but the source and nature of the dataset is not specified, neither here nor in the Methods section.

**Our Response**: This work reports the scRNA seq data of epidermal cells from human skin. This is now included.

7. P. 6 and Figure S2A - assuming that the other "publicly available human dataset " exists, as questioned above, it is strange that the authors simply list the top differentially expressed genes between the clusters in Figure S2A, and make no attempt to integrate this data with the data presented in Figure S1 (Belote et al.) to determine whether the different clusters identified in each separate dataset instead have shared features between datasets. In other words, does Cluster 0 in Figure S1 correlate highly with Cluster 1 (or any other numbered cluster) in Figure S2? It seems as if trying to answer this question, with the data and tools available to you, should be a greater priority than simply performing another assessment of melanocytic heterogeneity in zebrafish as the authors have done.

**Our Response:** Thank you for suggesting this. We have now mapped the clusters to data from Belote et al. and arrived at possible cluster identities across datasets and experiments. The same is included in the revised version as Fig S2D.

8. p.5, Figures S2A-C; the authors describe the use of EGAS00001002927, but I cannot find a reference to the manuscript or other resource that contains this dataset. The authors need to provide this information.

**Our Response:** This is included. Cheng et al 2018.

9. pp.6-7, experiment with forskolin (Fsk)-pulsed NHEMs: Which clusters correspond to which set of treated cells? (When initially described as clusters corresponding to the forskolin daily vs. alternate-day pulsed cells, which number clusters correspond to which treatment? Why isn't this stated up front in the text?

**Our Response:** Our apologies for omitting this inadvertently. The has now included and also indicated in Fig 1F.

10. P.6 (fsk-pulsed NHEM experiment) - of greater concern is the assumption made about fsk-pulsed NHEM, that their behavior will mimic the behavior of in vivo melanocytes and in vitro (cultured) MNT-1 cells from Malcov-Brog et al. (2018). Malcov-Brog et al. (2018) did not perform this experiment with NHEMs. The authors have not first independently validated the findings of Malcov-Brog et al. with NHEMs, namely that an alternate-day pulse with either UV or fsk results in greater pigmentation and expression of pigmentation genes, and a dampened oscillation of Mitf expression levels, as was shown with MNT-1 melanoma cells in Malcov-Brog et al. Hence the authors need to confirm similar basic findings with NHEM before they can build upon these conclusions with the single-cell expression analysis and clustering that they do in this manuscript.

**Our Response:** We have performed ICC and westernblot experiments NHEM to validate the observations made by the authors of Malcov-Brog et al. (2018) in MNT1. This is now included in Fig S3 & S4.

11. p. 11 - the "progressive pigmentation model" is not well-defined.

**Our Response:** We have given the details as well as the link to protocol.io towards this.

12. p. 12 - why can't NHEMs also be used to trace dynamics of state transitions?

**Our Response:** B16 gives the advantage of temporal resolution with synchronized pigmentation. This has been included in the revised version.

13. p. 13 - did Raja et al. (2020a) actually show that H3K27Ac selectively activated pigmentation genes?

**Our Response:** Yes.

14. p.15 - Principal component analysis, not "principle"

**Our Response:** Apologies for this inadvertent autocorrect. This has been changed.

15. p.15 - "all the three independent analyses described above" - please be specific and name the analyses.

**Our Response:** We have now elaborated the three analyses (DAR, DHAcR and TF enrichment).

16. In the colony analysis (p.10, Figure 3), how can the authors be sure they are capturing all of the cells optically when there is no specific cellular marker used?

**Our Response**: We trace the cells and colonies using bright field images and monitor them across days based on coordinates.
