# Supplementary material for: Distinct melanocyte subpopulations defined by stochastic expression of proliferation or maturation programs enable a rapid and sustainable pigmentation response
Source: PLoS Biol. 2024 Aug 20;22(8):e3002776. doi: 10.1371/journal.pbio.3002776 (PMC11364419; doi:10.1371/journal.pbio.3002776)
Supplement: Attachment — Submitted filename: Point By point response.docx [file pbio.3002776.s014.docx]

Dear Dr Natarajan,

Thank you for your patience while we considered your revised manuscript entitled "Stochastic segregation of proliferation and maturation programs into distinct subpopulations enables rapid and sustainable pigmentation response" for publication as a Research Article at PLOS Biology. This revised version of your manuscript has been evaluated by the PLOS Biology editors, the Academic Editor and one of the original reviewers.

Based on the review (attached below), we are likely to accept this manuscript for publication, provided you satisfactorily address the remaining points raised by Reviewer 2. Please also make sure to address the data and other policy-related requests stated below.

**Our Response:** We thank the Editorial team for the support throughout the review process. We are happy to note that the revision has elicited a positive response from the Reviewers.

In addition, we would like you to consider a suggestion to improve the title:

"Distinct melanocyte subpopulations defined by stochastic expression of either proliferation or maturation programs enable a rapid and sustainable pigmentation response to UV radiation"

**Our Response:** We thought over this and have arrived at the following title and we sincerely hope that this would be acceptable to the team. “**Distinct melanocyte subpopulations defined by stochastic expression of proliferation or maturation programs enable a rapid and sustainable Pigmentation response**”

As you address these items, please take this last chance to review your reference list to ensure that it is complete and correct. If you have cited papers that have been retracted, please include the rationale for doing so in the manuscript text, or remove these references and replace them with relevant current references. Any changes to the reference list should be mentioned in the cover letter that accompanies your revised manuscript.

**Our Response:** We have reviewed the list of references and found them to be accurate to the best of our knowledge. This includes the reference to Natarajan T, 2024, which was noted as missing by Reviewer 2.

We expect to receive your revised manuscript within two weeks.

To submit your revision, please go to <https://www.editorialmanager.com/pbiology/> and log in as an Author. Click the link labelled 'Submissions Needing Revision' to find your submission record. Your revised submission must include the following:
-  a cover letter that should detail your responses to any editorial requests, if applicable, and whether changes have been made to the reference list
-  a Response to Reviewers file that provides a detailed response to the reviewers' comments (if applicable, if not applicable please do not delete your existing 'Response to Reviewers' file.)
-  a track-changes file indicating any changes that you have made to the manuscript.

**Our Response:** We have included the revised version of the files as suggested.

NOTE: If Supporting Information files are included with your article, note that these are not copyedited and will be published as they are submitted. Please ensure that these files are legible and of high quality (at least 300 dpi) in an easily accessible file format. For this reason, please be aware that any references listed in an SI file will not be indexed. For more information, see our Supporting Information guidelines:
<https://journals.plos.org/plosbiology/s/supporting-information> 

**Our Response:** Our Supporting Information files are of required specification.

*Published Peer Review History*
Please note that you may have the opportunity to make the peer review history publicly available. The record will include editor decision letters (with reviews) and your responses to reviewer comments. If eligible, we will contact you to opt in or out. Please see here for more details:
<https://blogs.plos.org/plos/2019/05/plos-journals-now-open-for-published-peer-review/>

**Our Response:** We agree to have our peer review files available to all.

*Press*
Should you, your institution's press office or the journal office choose to press release your paper, please ensure you have opted out of Early Article Posting on the submission form. We ask that you notify us as soon as possible if you or your institution is planning to press release the article.

**Our Response:** Alright we will inform at the earliest.

*Protocols deposition*
To enhance the reproducibility of your results, we recommend that if applicable you deposit your laboratory protocols in [protocols.io](http://protocols.io/), where a protocol can be assigned its own identifier (DOI) such that it can be cited independently in the future. Additionally, PLOS ONE offers an option for publishing peer-reviewed Lab Protocol articles, which describe protocols hosted on [protocols.io](http://protocols.io/). Read more information on sharing protocols at <https://plos.org/protocols?utm_medium=editorial-email&utm_source=authorletters&utm_campaign=protocols>
**Our Response:** One of the protocols is submitted to [protocols.io](http://protocols.io/).and referenced.

Please do not hesitate to contact me should you have any questions.
Sincerely,

Ines Alvarez-Garcia, PhD
Senior Editor
PLOS Biology
[ialvarez-garcia@plos.org](mailto:ialvarez-garcia@plos.org)

------------------------------------------------------------------------

DATA POLICY:

Many thanks for submitting the files containing all individual quantitative observations that underlie the data summarized in the figures. However, we are still missing data from the following figures - please provide these:

Fig. 1B, D, E, F, G, I; Fig. 2A, C; Fig. 3A, D, E; Fig. 4B, D; Fig. 5B, D-G; Fig. S1A-E; Fig. S2A, D, E; Fig. S5C-E; Fig. S7A-D, F, G and Fig. S8A-F

Please also ensure that figure legends in your manuscript include information on WHERE THE UNDERLYING DATA CAN BE FOUND.

Please also ensure that your Data Statement in the submission system accurately describes where your data can be found.

**Please make sure the data you have deposited in GEO under the accession GSE233198 is made publicly available at this stage.

------------------------------------------------------------------------
**Our Response:** We have provided the corresponding data in the Supporting File named S3data.xlx. At the end of every figure a reference to this has also been provided.

We have begun providing public access to the referenced data.

CODE POLICY

Per journal policy, if you have generated any custom code during the course of this investigation, please make it available without restrictions. Please ensure that the code is sufficiently well documented and reusable, and that your Data Statement in the Editorial Manager submission system accurately describes where your code can be found.

Please note that we cannot accept sole deposition of code in GitHub, as this could be changed after publication. However, you can archive this version of your publicly available GitHub code to Zenodo. Once you do this, it will generate a DOI number, which you will need to provide in the Data Accessibility Statement (you are welcome to also provide the GitHub access information). See the process for doing this here: <https://docs.github.com/en/repositories/archiving-a-github-repository/referencing-and-citing-content>

**Our Response:** We have done this and the same has been indicated in the data availability section.

**Reviewer Response**

1. Introduction - "when the pigmentation process leads to a terminal differentiation state, which could reduce the likelihood of cell proliferation due to the burden on pigmentation (Bennett, 1983)". This construction remains awkward.

**Our Response:** We have reworded this to “In an earlier study, it was shown that dedifferentiation is associated with cell proliferation where most pigmented clones were small and most unpigmented clones large."

2. Introduction - "Manipulating the α-MSH pathway using a pharmacological activator," - unclear why there is the need for a comma after "activator"

**Our Response:** We have removed the punctuation as suggested.

3. At some point in the manuscript, the authors should explain how the side scatter population corresponds to cellular pigmentation (unless the explanation is present and I have overlooked it)

**Our Response:** We have now explicitly indicated the use of side scatter as a surrogate measure for pigment content in melanocytes and referenced the statement.

4. Results, newly added section on B16 cell immunofluorescence: "existence" rather than "existance".

**Our Response:** Thank you for bringing this to our notice we have now corrected the error.

5. Discussion: may want to refer to the "B16 progressive pigmentation model", just to keep the distinction between this model and the other experimental models used in the research (NHEM, MNT-1) fully distinct for the reader.

**Our Response:** We indicated the “B16” pigmentation model in the discussion.

6. Methods: should be "Alexa Fluor", not "Alexa Flour". Also, I think it is "principal components", not "principle components".

**Our Response:** Thank you for bringing this to our notice we have now corrected these errors.

7. Methods: Did the authors omit the description of techniques for the cellular and protein validation analysis of the MNT-1 cells?

**Our Response:** We have now included the western blot and immunocytochemistry methodology.

8. References: do not see the Natarajan et al. 2024 reference listed in the Bibliography.

**Our Response:** We checked this one is there under T Natarajan 2024.
